# Inference on Paleoclimate Change Using Microbial Habitat Preference in Arctic Holocene Sediments

**DOI:** 10.1038/s41598-017-08757-6

**Published:** 2017-08-29

**Authors:** Dukki Han, Seung-Il Nam, Ji-Hoon Kim, Ruediger Stein, Frank Niessen, Young Jin Joe, Yu-Hyeon Park, Hor-Gil Hur

**Affiliations:** 1 School of Earth Sciences and Environmental Engineering, Gwangju Institute of Science and Technology, Gwangju, 61005 Republic of Korea; 2 Korea Polar Research Institute, Incheon, 21990 Republic of Korea; 3 Petroleum and Marine Research Division, Korea Institute of Geosciences and Mineral Resources, 124 Gwahang-no Yuseong-gu, Daejeon, 34131 Republic of Korea; 4 Alfred Wegener Institute (AWI) Helmholtz Centre for Polar and Marine Research, Am Alten Hafen 26, Bremerhaven, 27568 Germany; 5 Department of Geosciences (FB5), Klagenfurter Str. 4, University of Bremen, 28359 Bremen, Germany; 6 Division of Earth Environmental System, Pusan National University, Busan, 609-735 Republic of Korea

## Abstract

The present study combines data of microbial assemblages with high-resolution paleoceanographic records from Core GC1 recovered in the Chukchi Sea. For the first time, we have demonstrated that microbial habitat preferences are closely linked to Holocene paleoclimate records, and found geological, geochemical, and microbiological evidence for the inference of the sulphate-methane transition zone (SMTZ) in the Chukchi Sea. In Core GC1, the layer of maximum crenarchaeol concentration was localized surrounding the SMTZ. The vertically distributed predominant populations of Gammaproteobacteria and Marine Group II Euryarchaeota (MG-II) were consistent with patterns of the known global SMTZs. MG-II was the most prominent archaeal group, even within the layer of elevated concentrations of crenarchaeol, an archaeal lipid biomarker most commonly used for Marine Group I Thaumarchaeota (MG-I). The distribution of MG-I and MG-II in Core GC1, as opposed to the potential contribution of MG-I to the marine tetraether lipid pool, suggests that the application of glycerol dibiphytanyl glycerol tetraethers (GDGT)-based proxies needs to be carefully considered in the subsurface sediments owing to the many unknowns of crenarchaeol. In conclusion, microbiological profiles integrated with geological records seem to be useful for tracking microbial habitat preference, which reflect climate-triggered changes from the paleodepositional environment.

## Introduction

The Arctic Ocean plays a key role in global ocean circulation and climate change^1–3^. Since global warming has been associated with the rapid reduction of Arctic sea ice, the debate on environmental change in the Arctic Ocean has become an issue of intense scientific interest. In this context, the Chukchi Sea and the adjacent Chukchi Borderland are of special importance, as the retreat in sea ice over the last several decades seems to be most dramatic in these areas in comparison to other marginal Arctic seas. The Chukchi Sea, extending from 66°N in the south to the edge of the Arctic Basin in the north, is connected to the North Pacific Ocean via the Bering Strait, which is the pathway for the inflow of the nutrient-rich, less-saline Pacific Water (i.e., the Bering Strait Inflow - BSI)^2^. With a present depth of approximately only 30–50 m, the Bering Strait formed a land bridge between Alaska and Siberia during the last glacial period owing to the lowered sea level^3, 4^. This lowered sea level led to a strongly limited or even interrupted influx of the BSI into the Arctic Ocean between approximately 80 to 11 ky BP, resulting in very different hydrographic and environmental conditions from those of today^4^.

In the Chukchi Sea, a high proportion of marine organic carbon is preserved in surface sediment as a result of elevated primary production owing to enhanced BSI and reduced sea-ice cover^5–8^. In contrast, terrigenous organic carbon is predominant in the Kara, Laptev, and Beaufort seas, as well as the central Arctic Ocean owing to the high supply of fluvial and coastal erosion sediment^5, 7^. Productivity in the central Arctic Ocean is low because of permanent sea ice cover, and subsequently a high relative abundance of terrigenous organic carbon in the surface sediments^7^. On geological time scales, the processes controlling organic carbon accumulation, such as primary production and terrigenous input, have changed significantly^5, 7^. During glacial periods the extended sea ice cover may have resulted in decreased primary production. On the other hand, the post-glacial sea-level rise may have triggered the onset of the BSI, reduced sea-ice cover, and subsequently increased primary production in the Chukchi Sea. However, the mechanisms underlying these processes, and their relationship to recent and past climate change, are not fully understood, and are thus subject of intense scientific and societal debate. In this context, high-resolution records of past climate and sea-ice conditions that extend beyond instrumental records—such the sediment cores we collected from the Chukchi Sea—may help to elucidate the complex Arctic Ocean-atmosphere-ice system and its role in the past, present, and future global climate, and to further improve climate models for a more accurate prediction of future climate change on Earth.

Microbial processes have been increasingly recognized as crucial for biogeochemical cycling in diverse environments, including oceans. Investigations of sub-seafloor microbiology in the world’s oceans have demonstrated that trapped and buried microbes in the subsurface sediments employ strategies of high biomass and low growth rates in response to sedimentation rate^9^. Parkes *et al*.^9^ also illustrated that microbial movements—such as mobility, growth, and diffusion in marine sediments—can determine whether microbes become buried within surface sediments or move into freshly deposited sediments. This means that a specific sediment layer may harbor a local microbial assemblage. An effective method to assess the effects of biogeochemical cycling on microbes in the ocean sub-seafloor is to characterize the sulphate-methane transition zone (SMTZ), as the anaerobic oxidation of methane (AOM) with complex and integrated microbial processes between anaerobic methanotrophs (ANME) and sulphate-reducing bacteria (SRB) occurs in this chemical zone^10^.

In general, microbial habitat preference can be an indication of the influence of environmental factors on the temporal and spatial distribution of microbial assemblages^11^. Recent studies on the Arctic subsurface biosphere^12, 13^ show that while the compositions of microbial assemblages in the Arctic subsurface sediments are similar to each other, their relative abundances are quite different. This discrepancy seems to be the result of geological distance and differences in sedimentary conditions between the shelf edge in the Beaufort Sea^13^ and the hydrothermal vent in the Arctic Mid-Ocean Ridge^12^. Such patterns of microbial distributions in the Arctic subsurface sediments can address the following questions: What is the nature of microbial habitat preference in the sub-seafloor? In addition, does the preference of microbial habitats explain geological events in the Arctic Ocean? Given the DNA-based survey for paleoclimatic changes (the so-called ‘paleome’) in Holocene sediments of the Black Sea^14–16^, the integrated research for tracking microbial habitats using DNA and paleoceanographic records in response to climate events (as reflected in changes in the BSI, sea-ice cover, and biological productivity in the Chukchi Sea after the reopening of the Bering Strait) deserves much consideration. The preservation of DNA over the course of geological time would be favourable in the subsurface sediment because of minimal influence on DNA degradation^17^. It has been reported that DNA is highly stable for an extremely long time at low temperatures under anoxic conditions^17^ (e.g., subsurface sediments), while the binding of DNA to clay particles appears to help prevent DNA degradation^18^. Additionally, the composition of microbial populations has been revealed by their membrane lipids in conjunction with DNA or RNA-based approaches in the sub-seafloor. Of the glycerol dibiphytanyl glycerol tetraethers (GDGTs), crenarchaeol has been utilized as a unique biomarker for mesophilic Crenarchaeota^19^, currently classified as Thaumarchaeota^20^ (herein Marine Group I); however, information on the biological source of crenarchaeol is still limited.

We investigated microbial assemblages in subsurface sediments, together with high-resolution paleoceanographic records preserved in sediment core ARA02B/01A-GC, in order to understand the processes driving microbial biogeographic patterns in the Arctic shelf sub-seafloor. The multidisciplinary approach—based on microbiological, geological, and geochemical proxies—will contribute to the understanding of this vast habitat and will help to further integrate microbial habitat preference and biogeochemical patterns in extremely cold Arctic environments.

## Results

**Figure 1:**
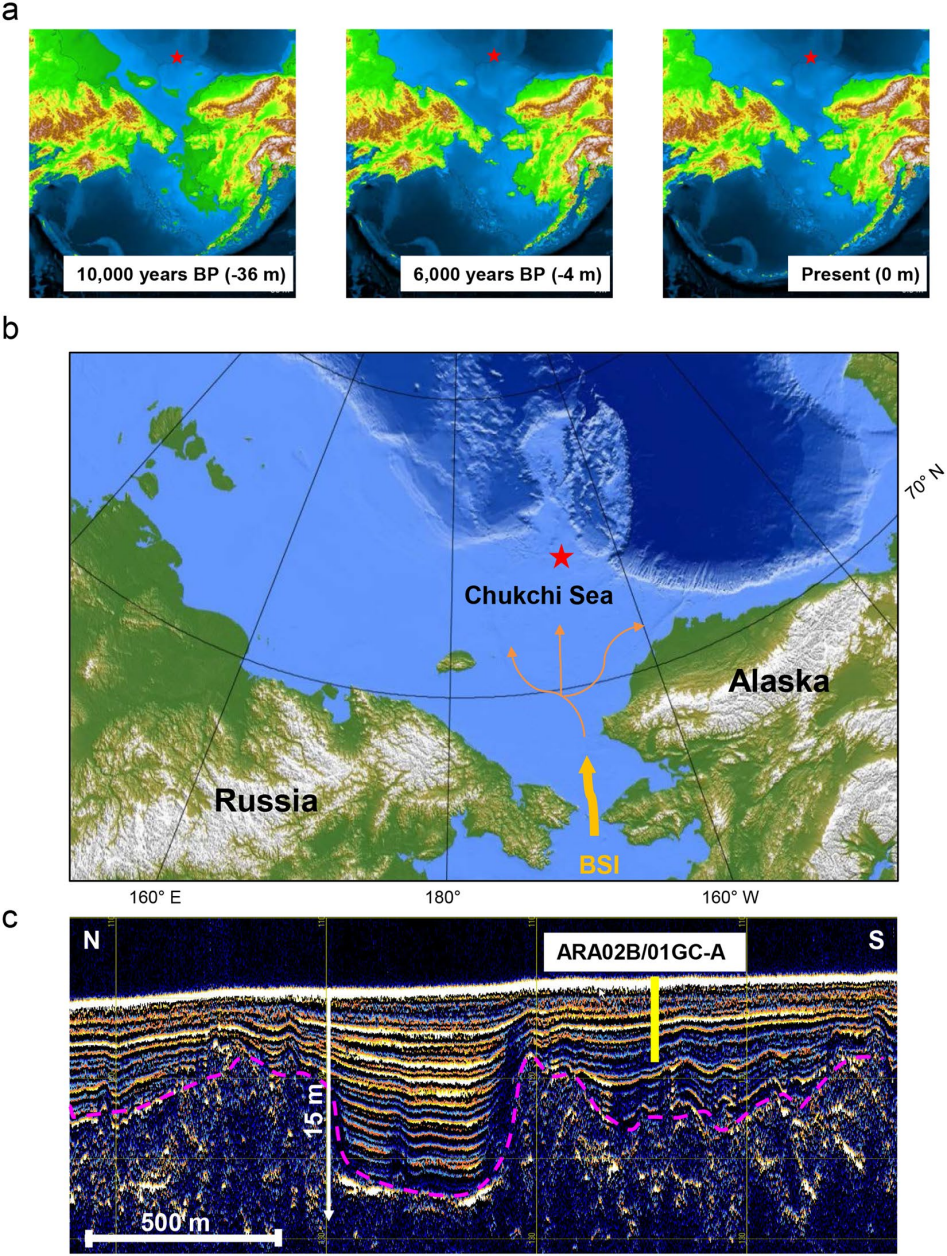
Geological history of the Chukchi Sea. (**a**) The post-glacial sea level rise during the Holocene (http://instaar.colorado.edu/QGISL/bering_land_bridge)^21^. (**b**) The modern bathymetric map for the research area (red colour star) under the influence of BSI. The bathymetric data was obtained from the IBCAO database^71^, and converted by the GMT 4.5.11 version (Generic Mapping Tools; http://gmt.soest.hawaii.edu). (**c**) The Parasound profile in the research area showing the coring position of the ARA02B/01A-GC (Core GC1). The profiling data was converted by the data processing software of the parametric hull-mounted system ATLAS HYDROGRAPHIC PARASOUND DS III-P70. Acoustic reflection pattern represents iceberg scouring (pink dotted line).

During recent years, several paleoceanographic studies have focused on the understanding of the interactions between BSI, sea-ice dynamics, and primary production in the Chukchi Sea since the postglacial re-opening of the Bering Strait^8, 21, 22^ (Fig. 1a). The sediment core ARA02B/01A-GC, hereafter referred to as Core GC1, was retrieved on the Chukchi Sea inner shelf during the Korean Ice Breaker Research Vessel (IBRV) *ARAON* Expedition in 2011. During the Holocene, the coring location of GC1 is strongly influenced by the BSI (Fig. 1b), whereas the study area had been intensively ploughed by icebergs during the last glacial period (Fig. 1c)^23^. Such depressions formed by grounding icebergs were acting as natural sediment traps for Holocene marine deposits. Core GC1 contains this sedimentary record with a high degree of temporal resolution (for site selection see “Methods”), and is thus suitable for detailed reconstruction of the post-glacial (Holocene) time period^8^.

### Holocene paleoclimate records of core ARA02B/01A-GC

**Figure 2:**
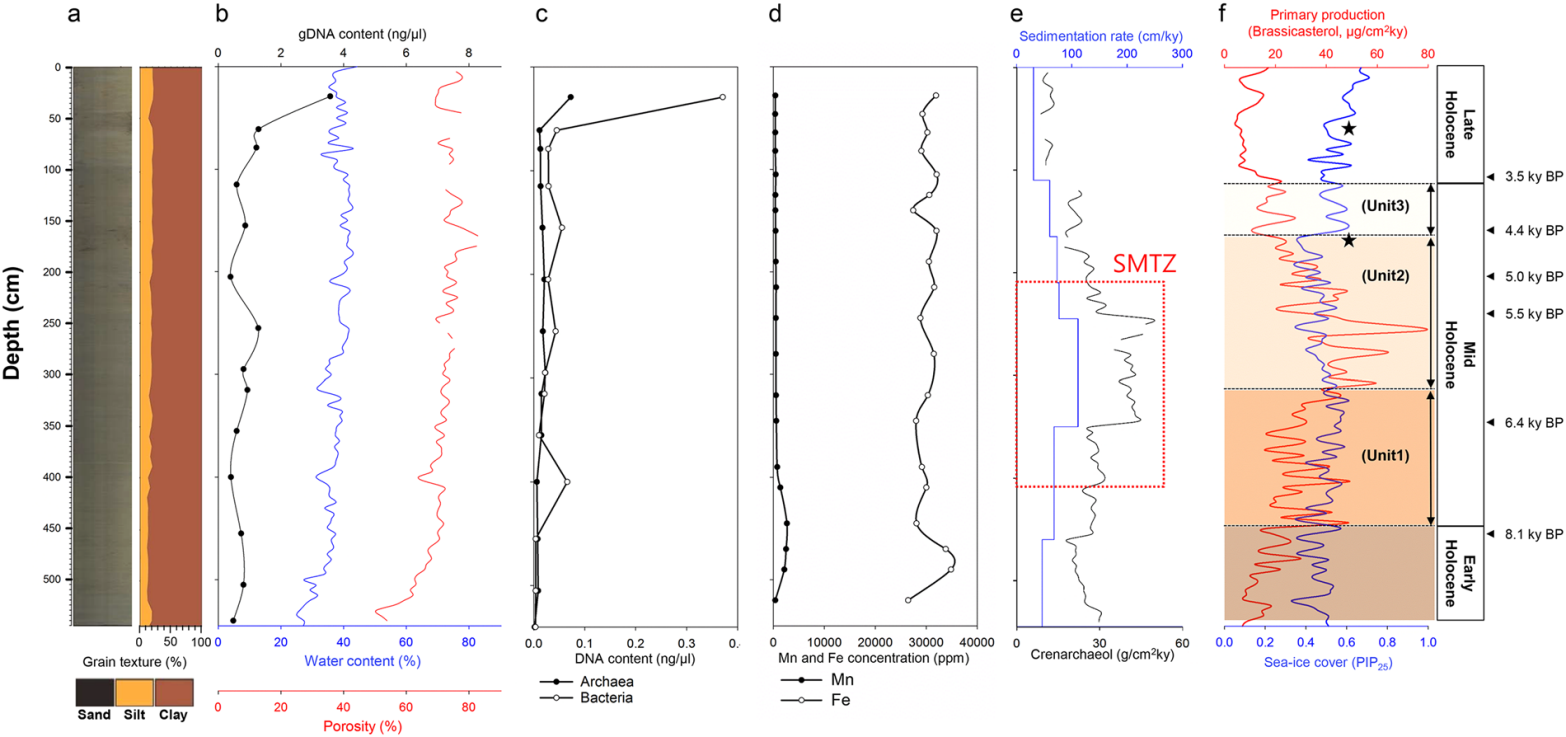
Downcore variation of sediment properties in the Arctic Holocene sediments. (**a**) Grain texture, (**b**) profiles of water content, porosity and gDNA, (**c**) distribution of both archaeal and bacterial DNAs, (**d**) iron (Fe) and manganese (Mn) elements, (**e**) sedimentation rate, accumulation rate of crenarchaeol, and (**f**) primary production and sea-ice cover changes in response to the Holocene climate trend in Core GC1. The black colored stars in (**f)** indicate the sea-ice expansion at approximately 4,500 and 2,000 years BP. The age model as well as the porosity in (**b**) and figures of both (**e** and **f**) were modified from Core GC1 dataset^8^. The accumulation rate of crenarchaeol in Core GC1 was calculated from the GDGTs dataset^67^ according to the mass accumulation rate (MAR; see in the documented dataset)^8^.

The sedimentary facies of Core GC1 are dominated by greyish olive green (5YG3/2) silty clay (Fig. 2a). The water content and porosity of Core GC1 show similar patterns throughout the sedimentary section (Fig. 2b), with quite constant values of 38.3 ± 2.8% and 73.0 ± 3.5%, respectively, in the upper 450 cmbsf. Below 450 cmbsf, both water content and porosity values continuously decrease with increasing depth of the core, implying less permeability of the silt-clay sediments in the lower part of Core GC1. With the exception of the uppermost section of Core GC1 (29 cmbsf; 3.57 ng/μl), genomic DNA (gDNA) content is less than 1.3 ng/μl (Fig. 2b), and shows a similar pattern in the down-core profile as both water content and porosity. The distribution patterns of both archaeal and bacterial DNA content also follow that of gDNA (Fig. 2c), and their sum is less than the gDNA.

Generally, the microbial biomass in non-hydrothermal sediments is correlated with depth-dependent factors such as age and porosity^9^, as well as with sedimentation rate^24^. Indeed, the gDNA profile of Core GC1 also follows the depth-dependent factors, with the exception of the uppermost layer representing oxic sediments. Of the gDNA, the extent of prokaryotic portion (archaeal and bacterial DNAs) is underestimated, possibly owing to the existence of eukaryotic and viral DNA, limited primer coverage, and/or DNA degradation. The profile of iron (Fe) and manganese (Mn) revealed the distinct Fe-Mn input during the early Holocene (Fig. 2d). According to the sedimentation rate of Core GC1, the amount of supplied and subsequently deposited sediments is highly variable during the Holocene^8^, reaching maximum values in the mid-Holocene between 245 and 345 cmbsf (Fig. 2e). Similarly, the accumulation rate of crenarchaeol followed sedimentation patterns (Fig. 2e). In general, the Holocene climate appears to be relatively stable, but the sudden and distinct cooling events were globally marked by polar cooling, tropical aridity, and major changes in atmospheric circulation^25^. Indeed, the Holocene cooling events were previously featured in the Northern Hemisphere at approximately 8,200, 4,500, and 2,000 years BP^8, 22, 26^. In Core GC1, sea-ice expansion periods at approximately 4,500 and 2,000 years BP were inferred from the variability of BSI, sea-ice cover, and primary production (Fig. 2f and Table 1).

**Table 1:** The Arctic Holocene climate record described by Core GC1^8^.

Early Holocene (~8,000 years BP^&^)	Mid Holocene			Late Holocene (3,700 ~ 0 years BP)
	Unit1 (8,000 ~ 6,200 years BP)	Unit2 (6,200 ~ 4,500 years BP)	Unit3 (4,500 ~ 3,700 years BP)	
Minimum sea-ice cover, Limited BSI^*^, Very low primary production	Sea-ice cover increase, BSI increase, Primary production increase	Minimum sea-ice cover, Maximum BSI, Maximum primary production	Sea-ice cover increase, High BSI, Primary production decrease	Maximum sea-ice cover, Low BSI, Low primary production

^&^BP: Before Present.

^*^BSI: Bering Strait Inflow.

### Microbial diversity and assemblage composition

**Figure 3:**
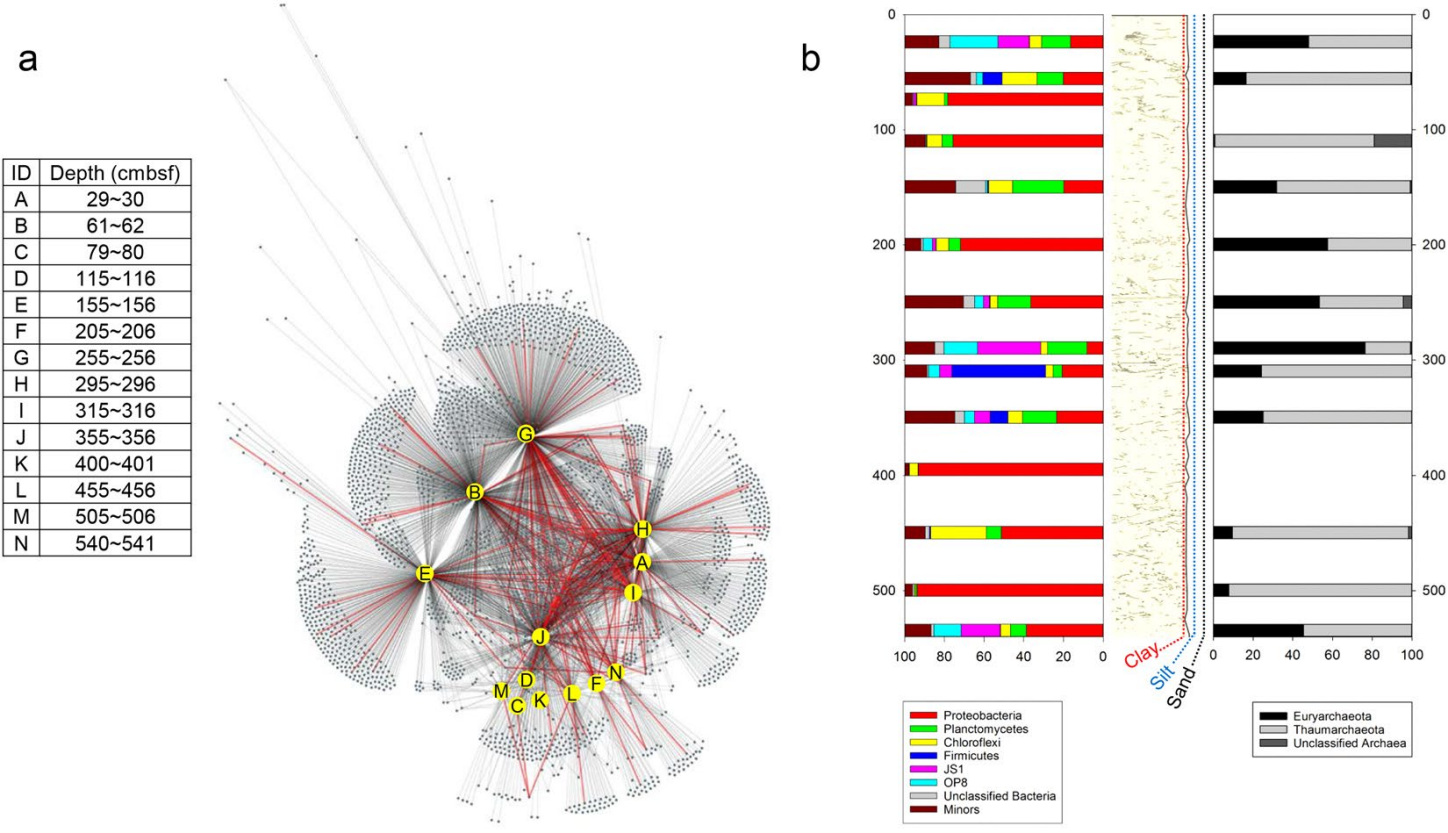
Microbial association network and assemblage composition. (**a**) Microbial OTU network, in which archaeal OTUs are connected with red colored lines. (**b**) Distribution of microbial populations in phylum level. Geologic column in (**b)** was drawn from X-ray images in Core GC1, and its grain size displayed with colored dotted lines was from the grain texture in Fig. 2a.

Fourteen horizons (A to N) were selected and sequenced from Core GC1. A total of 4,337 consensus operational taxonomic units (OTUs) were identified and visualized in the microbial association network (Fig. 3a). The microbial OTU network demonstrates that the number of bacterial OTUs (*n* = 4,211) overwhelmed the archaeal OTUs (*n* = 126), and that their OTU networks are similar to each other. The horizons A, B, E, G, H, I, and J had numerous OTUs, while the other horizons (C, D, F, K, L, M, and N) did not. Species richness for horizons C, D, F, K, L, M, and N was remarkably lower than for horizons A, B, E, G, H, I, and J (Table S1). In archaeal sequences, horizons C and K were removed owing to limited data (six sequences assigned into two OTUs in horizon C) and no archaeal DNA sequence (horizon K), and thus were not included in further non-biased taxonomic analyses. Such microbial diversity is also represented by the composition of microbial assemblages. For example, although bacterial sequences such as Proteobacteria (35.5%), Planctomycetes (12.8%), Chloroflexi (9.0%), Firmicutes (7.0%), JS1 (6.5%), and OP8 (6.1%); and archaeal sequences such as Thaumarchaeota (59.2%) and Euryarchaeota (39.4%) were the major taxonomic groups (phylum level) in Core GC1, their distribution varied in the less permeable silt-clay sediments (Fig. 3b). This variation is similar to the lack of a shared or closed node between the horizons in the microbial OTU network. Microbial assemblage compositions in horizons C, K, and M were relatively less complex in comparison to those of the other horizons, as evidenced by their lower values of species richness and evenness (Table S1).

### Microbial populations in the Holocene sediments

**Figure 4:**
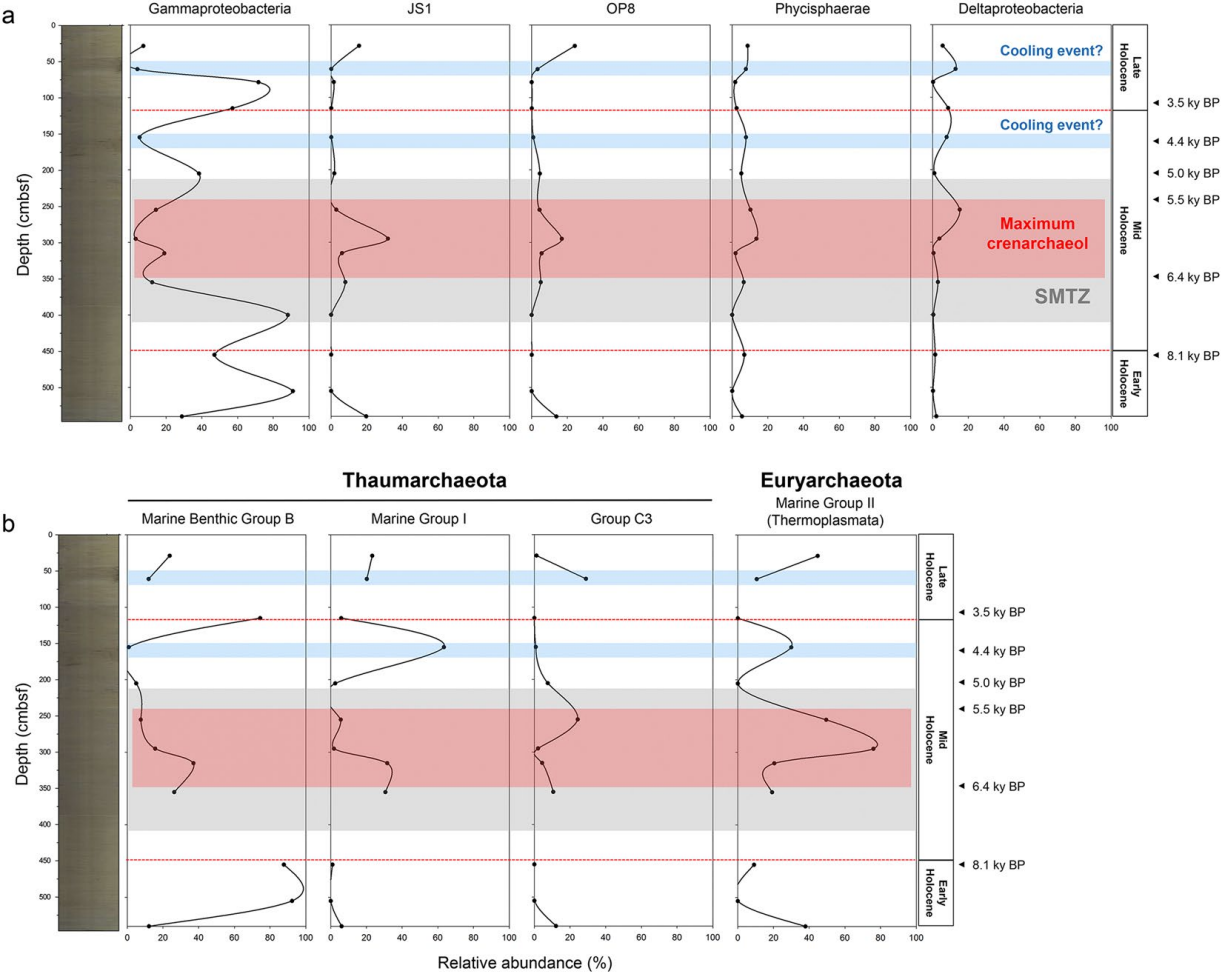
Distribution of major microbial populations under the Arctic Holocene. (**a**) Bacterial and (**b**) archaeal populations on the Holocene climate patterns projected by paleoclimate records in Fig. 2 and the SMTZ in Fig. S1.

Microbial assemblages in Core GC1 consist of few major populations (five bacteria and four archaea) with frequencies greater than 5% in the sum of sequences at the class level (Fig. 4 and Table S2), where their distributions are significantly independent along the deeper horizons (Table S3). Of the major bacterial groups (Fig. 4a), most sequences from the Proteobacteria are assigned to the gamma-subclass, one of the most active microbial groups in the subsurface sediments^27^. The distribution of Gammaproteobacteria is relatively variable compared to the other populations in Core GC1. For example, the relative abundance of Gammaproteobacteria is lower surrounding the layer of the SMTZ (as inferred from the sulphate-methane profile in Core ARA06C/01JPC; Fig. S1), where crenarchaeol levels or sedimentation rates are much higher than in the neighboring horizons. In general, the Gammaproteobacteria show the up-and-down variation (bimodal pattern) at the horizons representing cooling events and the maximum sedimentation rate (or maximum crenarchaeol).

The bacterial candidate division JS1^28^, recently known as Atribacter^29^, is an abundant phylotype in organic-rich environments associated with methane^30^. Bacterial sequences assigned as JS1 in the present study exhibit a distribution similar to that of candidate division OP8, first identified in sediments from the Obsidian Pool in Yellowstone National Park^31^. In contrast to the variation of Gammaproteobacteria, sequences of both JS1 and OP8 are intensified surrounding the layer of the SMTZ, which has the maximum crenarchaeol concentration. Distribution of both the Phycisphaerae and Deltaproteobacteria in Core GC1 exhibit similar patterns to the previously known SMTZ trend^10^. The relative abundances of the Phycisphaerae and Deltaproteobacteria seem to be intensified surrounding the SMTZ, but are more variable along the core depth. Most archaeal sequences (90.6%) were classified into three groups in the phylum Thaumarchaeota and one in the phylum Euryarchaeota (Fig. 4b). Of the three groups from the Thaumarchaeota, Marine Benthic Group B (MBG-B), now known as Lokiarchaeota^32^, has been detected in a variety of anoxic marine environments^30, 33–37^. The relative abundance of MBG-B in Core GC1 seems to be similar to that of the Gammaproteobacteria despite its limited data in horizons C (79–80 cmbsf) and K (400–401 cmbsf). Marine Group I Thaumarchaeota (MG-I), a chemolithoautotroph that oxidizes ammonia to nitrite^38^, was abundant (greater than 60%) in comparison to the featured sediment layer of the SMTZ, which has the maximum crenarchaeol concentration. Another group, Group C3 Thaumarchaeota, one of the thermophilic crenarchaeotal groups^39^, is a potential acetate consumer in marine sediments^40^. The distribution of Group C3 was variable in Core GC1 and not widespread in the methane-rich sediments below the SMTZ (herein the early Holocene, Fig. S1). In the Euryarchaeota, almost all sequences from the class Thermoplasmata are assigned to the order Thermoplasmatales, which is phylogenetically related to Marine Group II^41, 42^ (Fig. S2). The relative abundance of the Thermoplasmata in Core GC1 is highest surrounding the SMTZ compared to the methane-rich early Holocene sediments. In the present study, Gammaproteobacteria and Thermoplasmata are the most predominant populations, and their relative abundance shows an antiparallel distribution in Core GC1. The variation of Gammaproteobacteria and Thermoplasmata between the SMTZ and methane-rich sediments is similar to their global distribution patterns^43^.

**Figure 5:**
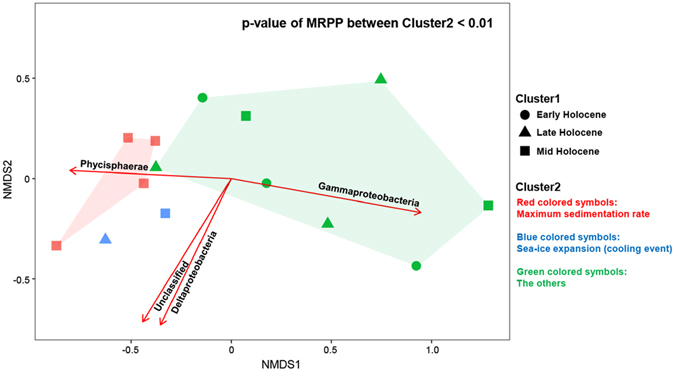
The NMDS ordination plot comparing bacterial assemblages (class level) in Core GC1. Each data point in the NMDS plot represents the bacterial assemblages identified from a single horizon. Arrows denote the most important populations explaining the separation pattern of the assemblages. Comparison using MRPP revealed a significant difference between the three groups (p < 0.01).

The relative abundance of major populations seems to be featured at the sediment layers of maximum sedimentation rate or sea-ice expansion (cooling event). This trend was estimated by further statistical analyses such as NMDS (Non-metric multidimensional scaling) and MRPP (multi-response permutation procedure). In the present study, the relative abundance of archaeal assemblages was not considered in the NMDS and MRPP for a statistical reason (empty values at 79–80 and 400–401 cmbsf). In Fig. 5, the NMDS ordination plot shows that bacterial assemblages in the layer of maximum sedimentation rate and sea-ice expansion are different from other assemblages, where a comparison using MRPP revealed a significant difference between the three groups (p < 0.01). Most notably, the difference in the composition of assemblages as detected by NMDS analyses is most significantly explained by the distribution of the Gammaproteobacteria (r^2^: 0.93, p = 0.001).

## Discussion

Recent studies of the Arctic deep-biosphere^12, 13^, as well as the present study, show that microbial assemblages are associated with geochemical traits and geological distance (geographical features/physical geography). For example, the composition of microbial assemblages at the shelf edge of the Beaufort Sea^13^ and in the hydrothermal vent systems of the Arctic Mid-Ocean Ridge^12^ are similar; however, they differ in their relative abundances of microorganisms. Such a discrepancy has also been observed in the current study of the Chukchi Sea. The sediment cores ARA02B/02GC and 03GC, obtained from the nearby Chukchi margin (increasingly farther north in the Chukchi Sea, respectively; Fig. S3), showed distinct water contents and DNA profiles in comparison to the sediments of the continental shelf (Core GC1; ARA02B/01A-GC in Fig. S3) in the Chukchi Sea. These sediment cores have also similar compositions of microbial assemblages, but differ in microbial distributions (Fig. S4), possibly owing to different sedimentary and environmental conditions from land as previously suggested on a global scale^44^.

Microbial interpretation based on paleoclimate records in subsurface sediments is a complicated process, but may help to guide future studies of the subsurface biosphere. One of the most important results of the present study is that the comprehensive records from Core GC1 elucidate the microbial habitat preference in the face of Holocene climate variability in the Arctic subsurface sediments. Integrated investigations of paleoclimate reconstruction could be effectively carried out in the Chukchi Sea shelf where the sedimentation is closely related to sea-ice dynamics, as well as to general oceanic climate factors such as terrigenous input, primary production, and hydrographic interaction between the water column and seafloor after the post-glacial re-opening of the Bering Strait. In other words, Core GC1 is geologically ideal for the investigation of the paleoclimate in the western Arctic Ocean owing to the exceptional preservation of buried organic matters and the relatively high sedimentation rate^8^. Moreover, the less permeable silt-clay sediment of the Core GC1 reflects environmental heterogeneity from lithological properties (e.g., physical properties, grain size, mineral composition, sedimentation rate, and porosity), as well as geochemical properties in subsurface sediments. Thus, the tracking of microbial habitat preference may reflect climate-triggered changes from Holocene marine deposits in the western Arctic Ocean because of the quality and quantity of the buried sedimentary organic carbons, as well as the source of organic matter.

Microbial habitats have been explained by ecological terms such as spatial scale, dispersal ability, ecological drift, diversification, and environmental selection^45^ in oceans worldwide^43, 46, 47^, as well as in the western Arctic Ocean^48–50^. In case of sub-seafloor environments, the spatial scale may play a key role in understanding microbial habitat preference. For example, without considering the vertical separation (spatial scale) of geological and geochemical traits, microorganisms would appear to be randomly distributed along Core GC1,and would appear to comprise one microbial habitat^45^ as shown in the microbial OTU network and assemblage composition (Fig. 3). However, when considering the habitat separation of Core GC1, the distributions of major microbial populations clearly differ in distinct horizons of the Holocene sediments, suggesting that the spatial scale in the sub-seafloor habitat would be controlled by sedimentation variability via environmental changes. Under the current climate conditions, the average sedimentation rate is estimated to be 0.5–1 cm/kyr^−1^ in the central Arctic Ocean (increasing toward the continental margins by >10 cm kyr^−1^), and regionally reaches the maximum value of >100 cm kyr^−1^ on the continental shelf^51^. At the location of Core GC1, sedimentation rate varied owing to sea-level rise. Consequently, the niche preferences of microbial assemblages in Core GC1 may be linked to the Holocene paleoenvironment. Most notably, the distribution of the Gammaproteobacteria seems to be associated with the variability of sedimentation and/or unfavourable bacterial growth under conditions of the sea-level rise or sea-ice expansion, which Gammaproteobacteria experienced the remarkable changes in sea-ice cover (2,000 and 4,500 years BP)^8^.

Microbial assemblages consist of generalist populations, which can thrive in a wide range of environmental conditions with varied resources, while specialists only thrive in a narrow range of environmental conditions with limited resources. In the microbial assemblages of Core GC1, the class Gammaproteobacteria is the most widespread group, the members of which are considered to be generalists. Indeed, unfavourable environmental conditions for Gammaproteobacteria can be explained by the relationship between microbial movement and sedimentation in the subsurface sediments. In general, microbes within surface sediment layers are sufficiently motile to keep up with the sedimentation rate,; however, their movement in deeper sediments would be considerably slowed, thereby exhibiting low growth rates owing to low energy flux^9^. Such microbes would have difficulty avoiding burial, except for in active hydrothermal vents where additional energy sources become available. The maximum rate of bacterial movement in the subsurface sediments has been assumed to be nearly 18 mm year^−1^ under favourable conditions^9^, unlike the Arctic Ocean. Moreover, given the slow microbial motility in the sub-seafloor^27^, microbial movement with fluid transport after burial is less likely to have occurred in the less permeable silt-clay sediments of Core GC1, with relatively stable porosity and water content.

Legacies of historical/geological events can leave lasting signatures on the geographic distribution of microbial assemblages^45^. For example, Knab *et al*.^52^, introduced microbial processes for the formation of the SMTZ, thereby linking the historic change between marine and brackish sediments with sea-level change in the Back Sea. The present study investigated the lasting imprint of the SMTZ on the Holocene paleoenvironment in the Chukchi Sea. At site GC1, the mid-Holocene climatic conditions, as indicated by the maximum sedimentation rate and intensified primary production^8^, may have provided an opportunity for the formation of the SMTZ in the given horizons. In the subsurface sediments, the availability of buried organic matter to deeper depths is strongly controlled by the sedimentation rate. Such buried organic matter would be subject to various microbial catalytic reactions to form methane. Consequently, it has been known that methane in the deep oceans is consumed through AOM by a microbial consortium using sulphate or metal oxides, such as manganese and iron, as electron acceptors^53^. However, although the SMTZ is generally associated with the typical AOM that is accompanied by a consortium of ANME and SRB from the Deltaproteobacteria, the scarcity of known methanogens in the methane-rich sediments has also been documented in the Pacific Ocean^30^. Indeed, the known mesophilic methanogens were absent or rare in SMTZ sediments in both the Chukchi Sea (the present study) and the Beaufort Sea^13^, implying the involvement of other processes for the AOM^13^. Of the major populations in Core GC1, for example, the bacterial candidate division JS1 and Crenarchaeota MBG-B have been suggested to play a role in unknown AOM processes in the sub-seafloor^10, 24, 30, 54^. Most notably, JS1 (the most abundant bacteria surrounding the SMTZ in Core GC1) is likely to play an ecological role in fermentation and syntrophy in low-energy environments^29^. The distribution of MBG-B in the lowermost part of the Fe-Mn enrichment layer in Core GC1 may be explained by the large amount of manganese and iron input during the early Holocene according to the possibility of manganese- and iron-dependent AOM^53^, as similarly observed in the Arctic Mid Ocean Ridge^12^. Indeed, the variation of major populations, including both JS1 and MBG-B, in Core GC1 exhibits overall similarity to the SMTZ sediments in Santa Barbara Basin^10^, suggesting that this type of microbial habitat with an unknown AOM process seems to exhibit a SMTZ signature. Given the assumed habitat preference of the bacterial candidate division JS1 and OP8, as well as MBG-B based on geochemical stratification, these microorganisms could be considered to be specialists for the SMTZ in Core GC1.

In the current study, the most abundant archaeal population within the layer of maximum crenarchaeol concentration in Core GC1 is Marine Group-II, belonging to Euryarchaeota (MG-II), rather than MG-I, which has been known to be a major player in the production of crenarchaeol. Both MG-I and MG-II are usually predominant archaeal taxa in oceans^55^. If MG-I was the only source of crenarchaeol in response to temperature change in oceans^56^, MG-I could be considered to be a generalist that tolerates the habitat change from seawater to subsurface sediments after deposition, and that survives in a sub-seafloor habitat^57^. The sediment lineages MG-I archaea, which are phylogenetically distinct from planktonic MG-I, have been reported at the Arctic Ocean and the Peru Basin^12, 36^. To explain our finding of this scenario, the sub-seafloor habitat would be more ideal for MG-II than MG-I, and/or the DNA of MG-I would be preferentially degraded in comparison to MG-II. On the other hand, it is reasonable to consider another possibility for the origin and fate of crenarchaeol, such as the synthesis of crenarchaeol by MG-II^55^, and/or the recycling of this compound by benthic archaea^58, 59^. Although the sources of crenarchaeol cannot be exclusively explained by the relative distribution of the sedimentary archaea, it is possible to formulate hypotheses regarding crenarchaeol^55, 56, 60–63^. Together with the latter assumptions, the distribution of MG-I and MG-II in Core GC1 seems to support the idea that the exclusive contribution of MG-I to the marine tetraether lipid pool needs to be carefully constrained to ensure the reliability of the GDGT-based proxy.

It is also worth noting that tracking microbial habitat preference with DNA-based techniques should consider both living and dead populations. However, the determination of the precise populations that are active in the Arctic subsurface habitat is very difficult. For example, minor populations in the microbial assemblages could be overestimated as a result of the misdetection of metabolically inactive populations; however, major populations are less likely to be affected by such an overestimation in comparison to minor populations. Thus, the present study focused on the habitat preferences of the major populations in response to environmental heterogeneity, rather than a full-diversity survey of the subsurface sediments. In addition, other potential factors, such as DNA degradation and contamination, need to be carefully considered in DNA-based analyses. In the present study, we assumed that the preservation of DNAs and/or microbes during the Holocene would be favourable in subsurface sediments, thereby eliminating DNA degradation and/or contamination as a factor. It is well known that DNA molecules are highly stable for an extremely long time at low temperatures in ionic and anoxic conditions^17^, which are similar environmental conditions to subsurface sediments far from hydrothermal vents. Moreover, the binding of DNA to clay particles seems prevent DNA degradation^18^. Considering the sampling site of Core GC1, which mostly consists of silt and clay particles (see grain texture in Fig. 2a), DNA molecules could be stably preserved. However, it has been reported that natural contamination by groundwater seepage or fluid transport can disturb DNA in the subsurface sediment prior to sampling^17^. Given the columnar section of the inside GC core, with no continuous microcrack (Fig. 3b), natural contamination is not likely to have occurred in the present study. Taken together, the potential errors caused by the overestimation of minor populations, DNA degradation and contamination would be less of an issue in Core GC1, although we are not ruling out this possibility.

For the first time, we have demonstrated that microbial habitat preferences are closely linked to Holocene paleoclimate records, and found geological, geochemical, and microbiological evidence for the inference of the SMTZ in the Chukchi Sea. The present study highlights Arctic microbial habitat preference via an analysis of high-resolution paleoceanographic records in the Chukchi Sea. Lithological and geochemical properties, as well as microbial habitat preference in Core GC1, suggest that sub-seafloor microbiota are a reflection of environmental heterogeneity over the geological period. Therefore, the multidisciplinary approach performed in the present study is an effective method, not only for microbial habitat tracking in the Arctic subsurface biosphere, but also to gain a better understanding of paleoclimate change.

## Methods

### Sample description

The coring station for ARA02B/01A-GC (i.e., Core GC1; 73°37.89′N, 166°30.98′W) was located at the Chukchi Sea continental shelf and carefully selected by both detailed bathymetric mapping and sub-bottom profiling (Parasound). Primary goal of this survey was to find an area with high sediment accumulation and to exclude sediment redeposition and/or erosion^8^. After recovery, Core GC1 was immediately sealed and stored under refrigeration on board. Fourteen sediment samples (each 2–4 g wet weight; for details see Table S4) were collected and freeze-dried for further microbiological study. Manganese and iron were analyzed from 20 different sediments (each 1 g dry weight; for details see Table S4). For the analysis of grain size, sediment samples (each 5 g wet weight) were collected at 10 cm depth intervals (for details see Table S5). The other sediment properties (water content, porosity, sedimentation rate, accumulation rate of crenarchaeol and brassicasterol, and sea-ice marker PIP_25_) were determined at 5 cm depth intervals, and some of them were retrieved from the documented dataset^8^ except for the water content and accumulation rate of crenarchaeol (Table S5).

### Lithological and geochemical properties

Sediments for the grain size analysis were sieved through a 63 μm sieve after washing, desalination and removal of organic matter with 10% H_2_O_2_, and then measured by using a laser granulometer (Sedigraph 5000D, Micromeritics, Norcross, GA). Afterward, particles were defined as sand (>63 μm diameter), silt (4.0 to 63 μm), and clay (<4.0 μm) size. Manganese and iron concentrations were measured by an atomic absorption spectrophotometer (ContrAA 300, Analytik Jena, Jena, Germany) after sample digestion using a microwave digestion system (MDS 2000, CEM, Matthews, NC). Water contents were measured through a freeze-drying. The other lithological traits (age, sediment rate, and porosity) and the biomarkers for primarily production (accumulation rate of Brassicasterol) and sea-ice cover (PIP_25_) were originated from the documented GC1^8^ data. The basic information on the use of these biomakers was previously descriebd^64–66^. The accumulation rate of crenarchaeol in Core GC1 was newly estimated in this study from the raw GDGT dataset^67^, and the GDGT analysis in the Arctic sediments was described elsewhere^68^. To feature SMTZ in Core GC1, the sulphate-methane profile was inferred from that of Core ARA06C/01JPC. The measure of sulphate and methane in Core ARA06C/01JPC as well as the correlation with Core GC1 were described in the supplementary information.

### DNA extraction and real-time quantitative PCR

gDNAs were extracted from the freeze dried sediments (each 0.25 g dry weight) using the MOBIO’s PowerSoil DNA extraction kit and PowerClean Pro DNA Clean-Up Kit (MOBIO, Carlsbad, CA) according to the manufacturer’s specifications. An additional purification was performed by a commercial column filled with polyvinylpolypyrrolidone (Zymo-Spin^TM^ IV-HRC, Zymo Research, Irvine, CA) to remove PCR inhibitors. The concentration of gDNAs was determined by using the Quant-iT PicoGreen dsDNA Reagent (Molecular Probes, Eugene, OR), and the gDNAs were further subjected to PCR-based approaches. Each content of archaeal and bacterial 16 S rRNA genes was measured by using a real-time quantitative PCR (qPCR) with the archaeal specific primer (F-arc, 5′-CAGCMGCCGCGGTAA-3′; R-arc, 5′-CCCGCCAATTCCTTTAAGTT-3′)^12^ and bacterial specific primer sets (F-bac, 5′-ACTCCTACGGGAGGCAGC-3′), R-bac (reverse, 5′-ATTACCGCGGCTGCTGG-3′)^69^. The quantification standard for calibration of the *C*_t_ (threshold cycle) of a target gene consisted of a dilution series of a known amount of archaeal DNA (*Halogranum salarium*) and bacterial DNA (*Escherichia coli*). The DNAs from *Halogranum salarium* and *Escherichia coli* were used as a control. Real-time qPCR for the target genes in samples and standards was performed in triplicates with the Rotor-Gene 3000 (Corbett Research, Mortlake, Australia). The PCR reaction was performed in a total volume 20 µl using KAPA SYBRH FAST qPCR Kit (Kapa Biosystems, MA, USA) according to the manufacturer’s specifications. PCR reactions for the archaeal 16 S rRNA gene were performed using DreamTaq^Tm^ Green PCR Master Mix (Fermentas, MD, USA) and the following conditions: initial denaturation at 94 °C for 15 min, followed by 30 cycles of denaturation at 94 °C for 15 s, annealing at 65 °C (for the archaeal 16 S rRNA gene) or 60 °C (for the bacterial 16 S rRNA gene) for 30 s, elongation at 72 °C for 30 s, and a final extension at 72 °C for 7 min. Specificity of amplicons was confirmed by melt-curve analyses of the PCR products. The quantification of archaea and bacteria in samples was determined from *C*_t_ values of standard curves.

### Micobial 16S rRNA gene sequencing and data processing

The Roche 454 GS FLX Plus System at Macrogen Inc. (Seoul, Rep. of Korea) was used to amplify microbial 16 S rRNA gene in the gDNAs using primers targeting the V5 to V9 regions, which cover 87% and 94% of all prokaryotes in the RDP^12^. The primers were V1-787F (5′-*CCATCTCATCCCTGCGTGTCTCCGACTCAG*-[X]-ATTAGATACCCNGGTAG-3′) and V9-1492R (5′-*CCTATCCCCTGTGTGCCTTGGCAGTCTCAG*-GNTACCTTGTTACGACTT-3′). The adaptor sequences are indicated in italics, and the specific sequences of 16 S rRNA regions are underlined. Symbol [*X*] denotes a 10 nucleotide-long barcode uniquely designed for each sample. PCR reactions were performed in triplicates using AccuPrime Pfx DNA polymerase (Invitrogen, Carlsbad, CA) and the following conditions: initial denaturation at 94 °C for 15 min, followed by 25 cycles of denaturation at 94 °C for 45 s, annealing at 52 °C for 45 s, elongation at 72 °C for 1 min, and a final extension at 72 °C for 7 min. PCR amplicons were purified using the Qiaquick PCR purification kit (Qiagen, Valencia, CA), and concentrations were measured by the Quant-iT PicoGreen dsDNA Reagent. The purified amplicons were mixed in equimolar amounts before pyrosequencing. All sequences can be accessed from the Sequence Read Archive at EMBL database, under accession number PRJEB18096.

Sequence analysis as well as the filtering and removal of noise from pyrosequencing data were performed using the MOTHUR program according to the protocol (http://www.mothur.org/wiki/454_SOP)^70^. In brief, sequencing dataset (81,141 reads) was used to calculate a distance matrix after the quality-filtering processes. Operational taxonomy units (OTUs) were clustered with a 97% similarity cutoff, and these OTUs were further used for the construction of microbial association network by the Cytoscape program. Taxonomic classification of the individual reads was achieved using the SILVA database in MOTHUR. The statistical difference in microbial assemblages at sediment layers of the maximum sedimentation rate and sea-ice expansion was illustrated by the NMDS (Non-metric multidimensional scaling) and MRPP (multi-response permutation procedure) analyses using the vegan package in R (www.r-project.org). NMDS of assemblage compositions employs multiple random starting configurations and selects the result with the lowest stress values. MRPP is further employed to examine the assemblage difference among samples.

## Electronic supplementary material

Supplementary information
