# Supplementary material for: Inference on Paleoclimate Change Using Microbial Habitat Preference in Arctic Holocene Sediments
Source: Sci Rep. 2017 Aug 29;7:9652. doi: 10.1038/s41598-017-08757-6 (PMC5575242; doi:10.1038/s41598-017-08757-6)
Supplement: Supplementary file 1 — Supplementary information [file 41598_2017_8757_MOESM1_ESM.pdf]

Inference on Paleoclimate Change Using Microbial Habitat Preference in Arctic Holocene Sediments

Dukki Han[a], Seung-Il Nam[b*], Ji-Hoon Kim[c], Ruediger Stein[d,e], Frank Niessen[d], Young Jin Joe[b], Yu-Hyeon Park[f], and Hor-Gil Hur[a*]

[a]School of Earth Sciences and Environmental Engineering, Gwangju Institute of Science and Technology, Gwangju 61005, Republic of Korea; [b]Korea Polar Research Institute, Incheon 21990, Republic of Korea; [c]Petroleum and Marine Research Division, Korea Institute of Geosciences and Mineral Resources, 124 Gwahang-no Yuseong-gu, Daejeon 34131, Republic of Korea; [d]Alfred Wegener Institute (AWI) Helmholtz Centre for Polar and Marine Research, Am Alten Hafen 26, Bremerhaven 27568, Germany; [e]Department of Geosciences (FB5), Klagenfurter Str. 4, University of Bremen, 28359 Bremen, Germany; [f]Division of Earth Environmental System, Pusan National University, Busan 609-735, Republic of Korea.

Supplementary Information

1. Multi-sensor core logging and sampling details of Core ARA06C/01JPC

After recovering the sediment cores, the multi-sensor core logging (MSCL) system (GEOTEK, UK) was used to measure the magnetic susceptibility (MS) at a 10 mm resolution. Variation of the MS in sediments is usually controlled by the amount of magnetic particles such as ferromagnetic minerals (magnetite, hematite, etc.)[1]. In contrast, biotic productivity such as opaline silica or calcium carbonate is less likely to affect magnetic matter in sediments. Thus, a MS profile can give information about the terrigenous material input. Furthermore, the MS profile is often used for inter-core correlation.

The sediment core ARA06C/01JPC, hereafter referred to as Core JPC1, was

recovered using the Jumbo Piston Corer during the 2015 IBRV ARAON expedition in the Chukchi Sea. The coring station of Core JPC1 is 3.2 km southeast of the GC1 site. Cores GC1 and JPC1 were correlated using the MS profiles. In the sedimentary sequence of Core JPC1, sulphate (SO_4^{2-}) concentrations were measured at depths of 20, 70, 120, 150, 230, 280, 330, 380, 430, 480, 530, 580, 630, and 680 cmbsf, and methane (CH_4) concentrations were measured at depths of 150, 300, 460, and 610 cmbsf. Pore fluid was extracted by Rhizone and was collected with 24 ml acid-prewashed syringes. Subsamples for the anion analysis were collected directly from the syringes into acid-prewashed Nalgene high-density polyethylene bottles after the extracted pore fluid was filtered through a 0.2 μm disposable polytetrafluoroethylene filter. Sulphate was analyzed by ion chromatography (ICS-1500, Dionex) with an AS-40 auto sampler at the Korea Institute of Geoscience and Mineral Resources (KIGAM). Reproducibility is better than 3%, as estimated by repeated measurements of standard analysis. For headspace gas analyses, a 3 ml sediment sample was taken with a cut-off 5 ml plastic syringe from the freshly exposed end of each core section and extruded into a 20 ml glass serum vial. Following the methods described before[2,3], 2 ml of saturated NaCl was added to each vial, which was then sealed with a 10 mm thick septum and a metal crimp cap. Headspace gas was extracted post cruise by heating the sediment samples at 60℃ for 30 min at KIGAM, following the procedure described before[4]. HS gases were injected into an Agilent Technologies 7890A gas chromatograph with flame ionization detector (FID) at KIGAM to analyze hydrocarbon composition. Reproducibility from repeated standard analysis was better than 5%.

The sulphate-methane transition zone (SMTZ) in Core JPC1 was determined from the downcore profile of SO_4^{2-} and CH_4 profiles. Based on the correlation of the MS profiles of cores GC1 and JPC1, the depth occurrence of the SMTZ layer of Core JPC1 was transferred to Core GC1 (Fig. S1).

2. Barcode information in the microbial 16S rRNA gene (V59 region) sequencing

barcode ATACGACGTA	29-30cmbsf
barcode TCACGTACTA	61-62cmbsf
barcode CGTCTAGTAC	79-80cmbsf
barcode TCTACGTAGC	115-116cmbsf
barcode TGTACTACTC	155-156cmbsf
barcode ACGACTACAG	205-206cmbsf
barcode CGTAGACTAG	255-256cmbsf
barcode TACGAGTATG	295-296cmbsf
barcode TACTCTCGTG	315-316cmbsf
barcode TAGAGACGAG	355-356cmbsf
barcode TCGTCGCTCG	400-401cmbsf
barcode ACATACGCGT	455-456cmbsf
barcode ACGCGAGTAT	505-506cmbsf
barcode ACTACTATGT	540-541cmbsf

3. Supplementary figures and tables

Figure S1. Correlation between cores ARA02B/01A-GC and ARA06C/01JPC based on magnetic susceptibility profiles, and sulphate and methane concentrations of Core JPC1. The Parasound profile in the research area showing coring positions of cores ARA02B/01A-GC and ARA06C/01JPC. The red correlation line highlights the reflector of same age in cores ARA02B/01A-GC and ARA06C/01JPC.

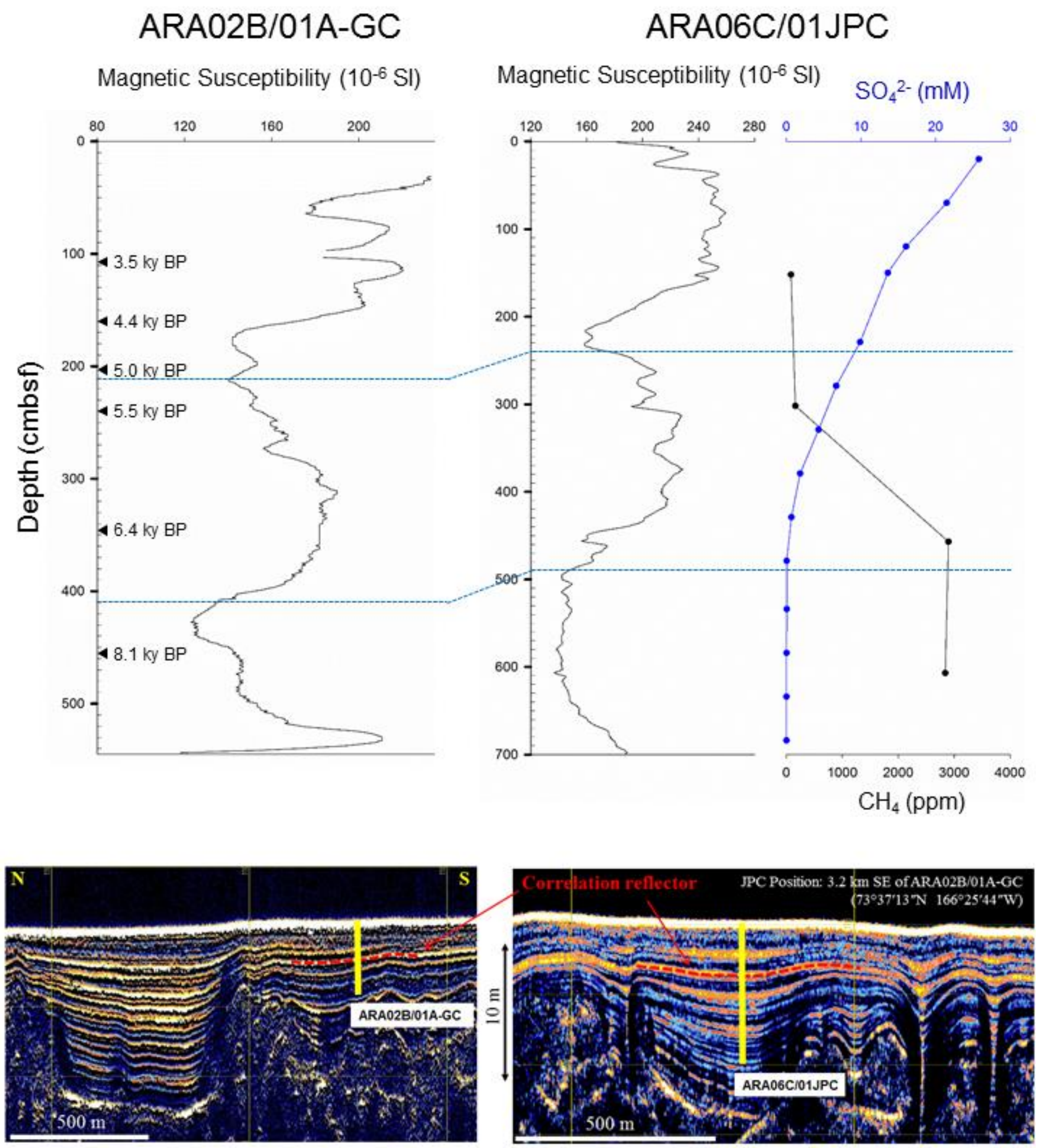

Figure S2. Phylogenetic analysis with representative sequences retrieved from major 34 OTUs of Thermoplasmatales (98.2% in the sum of Thermoplasmata sequences). These representative sequences were exported into MEGA5 software[5] using the Maximum Composite Likelihood method with 500 bootstrap tests to construct a phylogenetic tree along with seven archaeal 16S rRNA sequences (two MG-I, three MG-II, one *Methanobacterium* and one *E. coli* sequences) in GenBank and ENA databases.

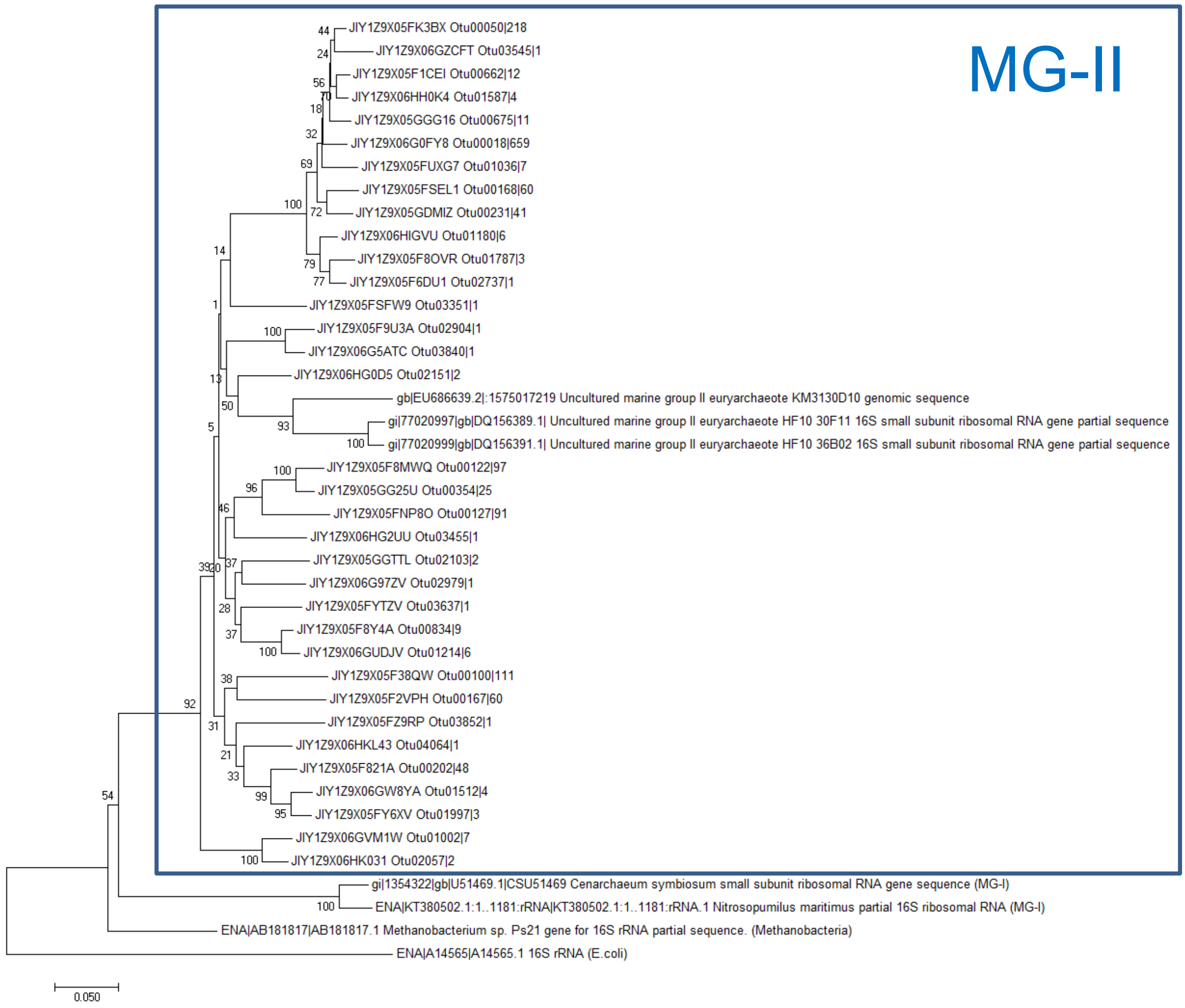

Figure S3. Sample description. A bathymetric chart and sub-bottom profile (SBP) were constructed to illustrate the oceanographic status of seafloor around the research area. The bathymetric data was obtained from the IBCAO database[6], and converted by the GMT software (Generic Mapping Tools; http://gmt.soest.hawaii.edu). The SBP120 system operates in a frequency range of 2.5-7 kHz, and is electronically stabilized for roll and pitch. Measured sub-bottom profiling data was converted by the data processing software of the EM122 multibeam system.

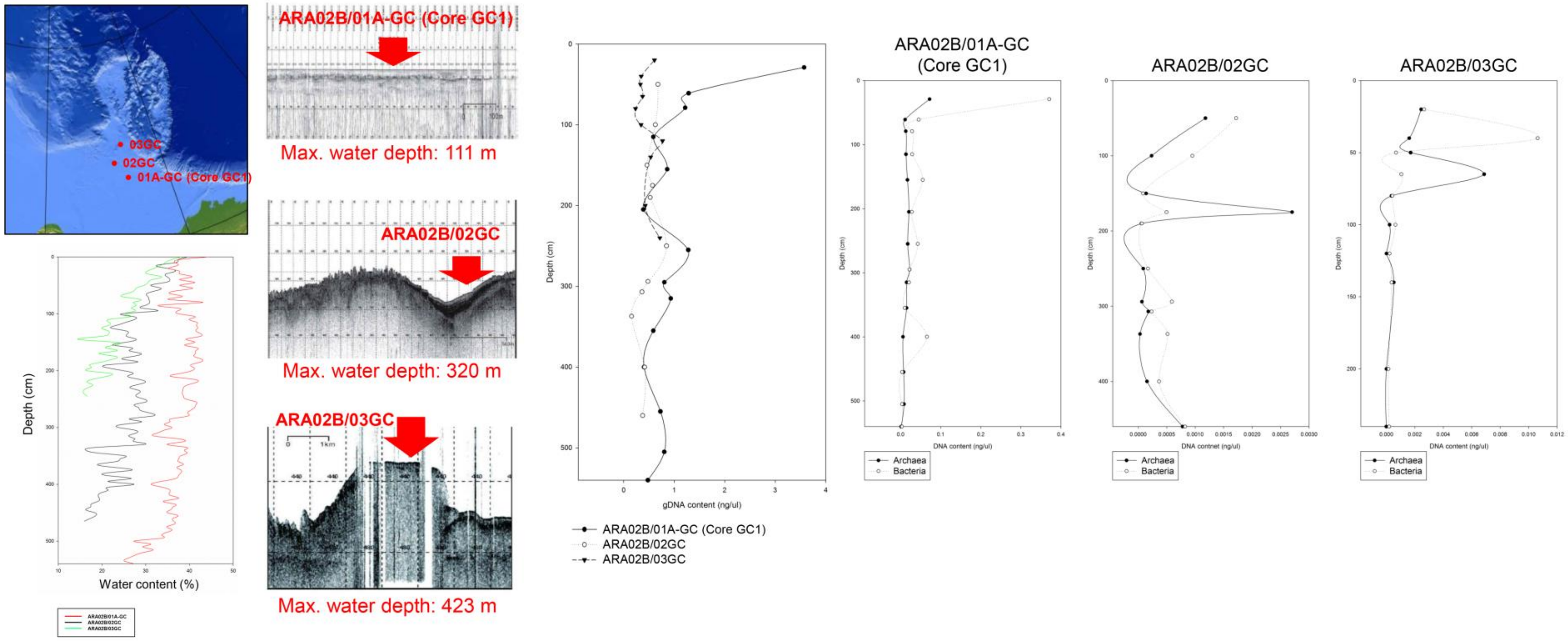

Figure S4. The composition of microbial assembles.

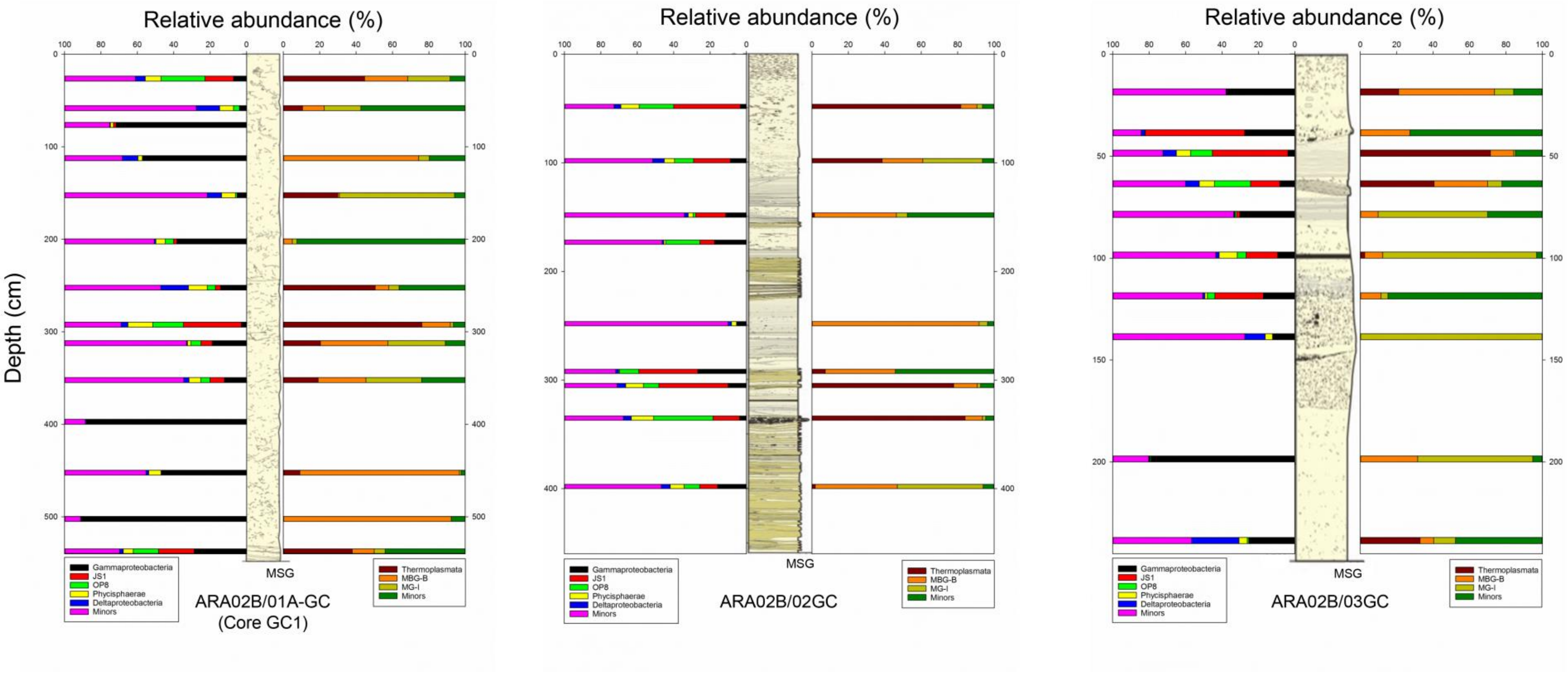

Table S1. Alpha diversity of microbial assemblages in Core GC1.

Sample (No. of reads)	Species richness		Species evenness	
	Chao	Ace	Shannon	Inverse Simpson
A (n=7,148)	950	951	4.4	16.5
B (n=9,525)	1830	1869	5.5	52.9
C (n=416)	32	36	1.8	3.9
D (n=1,839)	178	130	3.0	9.4
E (n=9,715)	1020	1046	5.4	60.6
F (n=3,173)	191	171	3.4	15.3
G (n=9,095)	3504	4775	6.3	167.6
H (n=8,155)	966	1198	4.0	9.1
I (n=8,241)	985	1324	2.7	4.5
J (n=6,587)	835	836	5.1	50.6
K (n=2,328)	39	41	1.2	1.8
L (n=7,039)	185	169	3.1	12.9
M (n=6,107)	72	72	2.0	3.7
N (n=1,773)	206	203	3.4	12.9

Table S2. Microbial populations in Core GC1.

Taxonomic classification			Depth (cmbsf)													
Domain	Phylum	Class	29	61	79	115	155	205	255	295	315	355	400	455	505	540
Archaea	Euryarchaeota	Halobacteria	1.0	1.4	nd*	0.0	1.7	0.0	2.9	0.3	0.8	5.5	nd	0.6	7.7	0.0
Archaea	Euryarchaeota	Methanomicrobia	2.2	3.7	nd	0.7	0.2	57.5	0.2	0.1	3.0	0.0	nd	0.0	0.0	7.6
Archaea	Euryarchaeota	Thermoplasmata	44.7	10.6	nd	0.0	29.9	0.0	49.5	76.0	20.4	19.2	nd	9.1	0.0	37.9
Archaea	Euryarchaeota	unclassified	0.0	0.9	nd	0.0	0.0	0.0	0.7	0.0	0.0	0.4	nd	0.0	0.0	0.0
Archaea	Thaumarchaeota	Group_C3	1.2	28.9	nd	0.0	0.8	7.5	24.3	2.1	4.4	10.6	nd	0.0	0.0	12.1
Archaea	Thaumarchaeota	Marine_Benthic_Group_A	0.0	3.7	nd	0.0	0.2	0.0	0.2	0.0	0.0	0.0	nd	0.0	0.0	0.0
Archaea	Thaumarchaeota	Marine_Benthic_Group_B	23.7	11.9	nd	74.5	0.8	5.0	7.5	15.6	37.1	26.3	nd	87.7	92.3	12.1
Archaea	Thaumarchaeota	Marine_Group_I	23.2	20.2	nd	5.8	63.4	2.5	5.6	1.8	31.6	30.6	nd	0.9	0.0	6.1
Archaea	Thaumarchaeota	Miscellaneous_Crenarchaeotic_Group	3.7	15.1	nd	0.0	2.1	27.5	4.6	3.4	2.5	7.5	nd	0.0	0.0	24.2
Archaea	Thaumarchaeota		0.2	3.2	nd	0.0	0.0	0.0	0.0	0.0	0.3	0.0	nd	0.0	0.0	0.0
Archaea	unclassified_archaea		0.0	0.5	nd	19.0	0.8	0.0	4.4	0.7	0.0	0.0	nd	1.7	0.0	0.0
			**100.0**	**100.0**		**100.0**	**100.0**	**100.0**	**100.0**	**100.0**	**100.0**	**100.0**		**100.0**	**100.0**	**100.0**
Bacteria	Acidobacteria	Acidobacteria	0.4	4.7	0.0	3.6	7.2	0.3	2.3	0.4	0.0	0.9	0.0	0.3	0.4	0.0
Bacteria	Acidobacteria	Holophagae	0.6	1.7	0.2	0.0	0.7	0.0	1.2	0.4	0.1	0.0	0.0	0.0	0.0	0.0
Bacteria	Acidobacteria	Subgroup_22	0.0	0.3	0.0	0.4	0.1	0.0	0.5	0.0	0.0	0.0	0.0	0.0	0.0	0.0
Bacteria	Acidobacteria	Subgroup_26	0.0	0.2	0.0	0.0	0.0	0.0	0.2	0.0	0.0	0.0	0.0	0.0	0.0	0.0
Bacteria	Acidobacteria	-	0.0	0.1	0.0	0.0	0.0	0.0	0.1	0.0	0.0	0.0	0.0	0.0	0.0	0.0
Bacteria	Actinobacteria	Acidimicrobiia	0.1	3.8	0.5	0.0	0.8	0.0	1.0	0.2	0.0	0.3	0.1	0.0	0.0	0.1
Bacteria	Actinobacteria	Actinobacteria	0.0	0.0	0.0	0.8	0.1	1.5	0.0	0.0	0.0	0.4	0.9	0.3	0.5	0.4
Bacteria	Actinobacteria	Coriobacteriia	0.0	0.0	0.0	0.0	0.0	0.0	0.0	0.0	0.0	0.0	0.0	0.0	0.0	0.0
Bacteria	Actinobacteria	OPB41	0.6	1.3	0.0	0.3	0.4	1.6	0.3	1.0	0.0	0.5	0.0	3.8	0.0	1.8
Bacteria	Actinobacteria	Thermoleophilia	0.8	0.6	0.5	1.1	1.2	0.1	0.1	0.1	0.2	0.7	0.0	3.3	1.3	0.1
Bacteria	Actinobacteria	-	0.0	0.2	0.0	0.0	0.1	0.1	0.2	0.0	0.0	0.0	0.0	0.0	0.0	0.0

Bacteria	Aquificae	Aquificae	0.0	0.0	0.0	0.9	0.0	0.0	0.0	0.0	0.0	0.0	0.0	0.0	0.0	0.2
Bacteria	Armatimonadetes	Chthonomonadetes	0.0	0.0	0.0	0.0	0.0	0.0	0.0	0.0	0.0	0.0	0.0	0.0	0.0	0.0
Bacteria	Armatimonadetes	-	0.1	0.1	0.0	0.0	0.2	0.2	0.1	0.4	0.0	0.1	0.0	0.0	0.0	1.0
Bacteria	BD1-5	-	0.0	0.6	0.0	0.0	0.7	0.0	0.6	0.2	0.0	0.1	0.0	0.0	0.0	0.0
Bacteria	BHI80-139	-	0.0	0.1	0.0	0.0	0.2	0.5	0.0	0.5	0.2	0.4	0.0	0.1	0.0	0.2
Bacteria	Bacteroidetes	BD2-2	0.2	0.7	0.0	0.0	0.2	0.0	2.0	0.1	0.0	0.0	0.0	0.4	0.0	0.4
Bacteria	Bacteroidetes	Bacteroidia	0.0	0.0	0.0	0.0	0.0	0.0	0.2	0.0	0.0	0.0	0.0	0.0	0.0	0.0
Bacteria	Bacteroidetes	Cytophagia	0.0	0.3	0.0	0.4	0.1	0.0	0.9	0.0	0.0	0.0	0.0	0.0	0.0	0.0
Bacteria	Bacteroidetes	Flavobacteriia	0.0	0.9	1.0	0.0	0.0	0.9	4.5	0.0	0.0	0.3	0.0	0.1	1.0	0.1
Bacteria	Bacteroidetes	SB-1	0.0	0.0	0.0	0.0	0.0	0.0	0.2	0.0	0.0	0.1	0.0	0.0	0.0	0.0
Bacteria	Bacteroidetes	Sphingobacteriia	0.0	0.1	0.0	0.8	0.0	0.0	0.6	0.0	0.0	0.1	0.0	0.0	0.1	0.4
Bacteria	Bacteroidetes	VC2.1_Bac22	0.0	0.1	0.0	0.0	0.0	0.0	0.1	0.0	0.0	0.0	0.0	0.0	0.0	0.0
Bacteria	Bacteroidetes	-	0.0	0.0	0.0	0.0	0.0	0.0	0.5	0.0	0.0	0.0	0.0	0.2	0.0	0.0
Bacteria	Bacteroidetes	vadinHA17	0.0	0.0	0.0	0.0	0.0	0.0	0.2	0.0	0.0	0.0	0.0	0.0	0.0	0.0
Bacteria	Candidate_division_BRC1	-	0.6	0.2	0.0	0.0	0.8	0.2	1.5	0.3	0.0	0.3	0.0	0.1	0.0	0.2
Bacteria	Candidate_division_JS1	-	15.6	0.1	1.5	0.1	0.1	1.8	2.9	31.8	6.1	8.0	0.0	0.0	0.0	19.6
Bacteria	Candidate_division_KB1	Unknown_Class	0.0	0.0	0.0	0.0	0.0	0.0	0.0	0.0	0.0	0.0	0.0	0.0	0.0	0.0
Bacteria	Candidate_division_KB1	-	0.0	0.0	0.0	0.0	0.0	0.0	0.0	0.1	0.0	0.0	0.0	0.0	0.0	0.0
Bacteria	Candidate_division_OD1	-	1.4	1.8	0.0	0.0	0.9	0.0	0.7	0.7	0.4	1.5	0.0	0.1	0.0	0.0
Bacteria	Candidate_division_OP11	-	0.4	0.5	0.7	0.0	0.1	1.1	0.2	2.2	0.2	3.7	0.0	0.0	0.0	2.2
Bacteria	Candidate_division_OP3	-	0.1	0.2	0.7	0.0	0.8	0.0	0.1	0.4	0.1	0.4	0.0	0.0	0.0	0.0
Bacteria	Candidate_division_OP8	-	24.2	3.3	0.0	0.1	0.9	4.6	4.4	16.9	5.6	5.1	0.0	0.0	0.0	13.8
Bacteria	Candidate_division_SR1	-	0.0	0.2	0.0	0.0	0.0	0.0	0.0	0.0	0.0	0.0	0.0	0.0	0.0	0.0
Bacteria	Candidate_division_TM7	-	0.0	0.0	0.0	0.0	0.0	0.0	0.1	0.0	0.0	0.0	0.0	0.0	0.0	0.0
Bacteria	Candidate_division_WS3	Unknown_Class	0.0	0.0	0.0	0.0	0.0	0.0	0.0	0.0	0.0	0.0	0.0	0.0	0.0	0.1
Bacteria	Candidate_division_WS3	-	0.3	1.8	0.0	0.0	0.4	0.0	2.4	0.3	0.1	0.2	0.0	0.0	0.0	0.3
Bacteria	Candidate_division_WS6	-	0.0	0.0	0.0	0.0	0.0	0.0	0.0	0.0	0.0	0.0	0.0	0.0	0.0	0.0

Bacteria	Chlamydiae	Chlamydiae	2.1	1.7	0.2	0.8	2.9	0.0	0.2	0.6	0.3	2.5	0.0	0.3	0.0	0.1
Bacteria	Chlorobi	Chlorobia	0.1	0.1	0.0	0.0	0.0	0.0	0.1	0.0	0.0	0.0	1.0	0.0	0.0	0.0
Bacteria	Chlorobi	Ignavibacteria	0.0	1.5	0.0	0.0	0.4	0.0	1.1	0.0	0.0	0.0	0.0	0.0	0.0	0.0
Bacteria	Chloroflexi	Anaerolineae	3.1	11.1	0.0	0.0	1.6	2.7	3.0	1.6	1.0	5.0	1.7	0.0	0.0	1.0
Bacteria	Chloroflexi	Ardenticatenia	0.0	0.0	0.0	0.0	0.0	0.0	0.0	0.0	0.0	0.0	0.0	0.0	0.0	0.0
Bacteria	Chloroflexi	Caldilineae	0.0	0.0	0.0	0.0	0.0	0.0	0.0	0.0	0.0	0.0	0.0	0.0	0.0	0.0
Bacteria	Chloroflexi	Dehalococcoidia	0.6	0.4	0.2	0.1	1.1	1.4	0.4	0.9	0.4	0.8	0.0	0.4	0.0	1.6
Bacteria	Chloroflexi	Gitt-GS-136	0.0	0.0	0.0	0.0	0.0	0.0	0.0	0.0	0.0	0.0	0.0	0.0	0.0	0.0
Bacteria	Chloroflexi	JG30-KF-CM66	0.4	1.3	0.0	0.1	4.3	0.3	0.1	0.0	0.0	0.1	0.1	0.5	0.0	0.3
Bacteria	Chloroflexi	KD4-96	0.0	0.2	0.0	0.0	0.0	0.0	0.0	0.0	0.0	0.0	0.0	0.0	0.0	0.0
Bacteria	Chloroflexi	MSB-5B2	0.7	0.0	0.0	0.0	0.0	0.2	0.0	0.1	0.3	0.1	0.0	0.0	0.0	0.0
Bacteria	Chloroflexi	S085	0.9	3.9	13.2	7.4	4.3	1.7	0.1	0.7	2.0	1.1	2.5	27.1	0.6	0.8
Bacteria	Chloroflexi	SAR202_clade	0.0	0.0	0.5	0.0	0.6	0.0	0.0	0.0	0.0	0.0	0.0	0.0	0.0	0.1
Bacteria	Chloroflexi	TK10	0.0	0.1	0.0	0.0	0.0	0.0	0.0	0.0	0.0	0.0	0.0	0.0	0.0	0.0
Bacteria	Chloroflexi	-	0.2	0.6	0.0	0.0	0.1	0.0	0.2	0.2	0.0	0.2	0.0	0.0	0.0	1.2
Bacteria	Cyanobacteria	Chloroplast	0.1	0.0	0.0	0.0	0.0	0.0	0.3	0.6	0.1	0.0	0.0	0.0	0.0	4.0
Bacteria	Cyanobacteria	Cyanobacteria	0.0	0.0	0.0	0.0	0.0	0.0	0.0	0.0	0.0	0.0	0.0	0.0	0.0	0.0
Bacteria	Cyanobacteria	ML635J-21	0.0	0.0	0.0	0.0	0.0	0.0	0.1	0.0	0.0	0.0	0.0	0.0	0.0	0.0
Bacteria	Deferribacteres	Deferribacteres	0.1	0.2	0.0	0.1	0.4	0.0	0.8	0.3	0.0	0.3	0.0	0.0	0.0	0.1
Bacteria	Deinococcus-Thermus	Deinococci	0.8	0.6	0.0	0.1	2.0	0.0	0.1	0.8	0.3	0.8	0.0	0.6	0.0	0.6
Bacteria	Elusimicrobia	Elusimicrobia	0.0	0.0	0.0	0.0	0.0	0.0	0.0	0.0	0.0	0.0	0.0	0.0	0.0	0.0
Bacteria	Fibrobacteres	Fibrobacteria	0.0	0.0	0.0	0.0	0.0	0.0	0.0	0.0	0.0	0.1	0.0	0.0	0.0	0.0
Bacteria	Firmicutes	Bacilli	0.1	0.1	0.0	0.1	0.2	0.1	0.0	0.0	47.1	0.3	0.2	0.2	0.0	0.0
Bacteria	Firmicutes	Clostridia	0.1	9.5	0.2	0.1	0.3	0.1	0.4	0.0	0.2	8.7	0.0	0.4	0.0	0.3
Bacteria	Firmicutes	OPB54	0.0	0.0	0.0	0.0	0.0	0.0	0.0	0.0	0.0	0.0	0.0	0.0	0.0	0.0
Bacteria	Firmicutes	-	0.0	0.0	0.0	0.0	0.0	0.0	0.0	0.0	0.0	0.0	0.0	0.0	0.0	0.0
Bacteria	GOUTA4	-	0.0	0.0	0.0	0.0	0.0	0.0	0.0	0.0	0.0	0.2	0.0	0.0	0.0	0.0

Bacteria	Gemmatimonadetes	Gemmatimonadetes	0.1	5.0	0.0	0.0	1.3	0.0	1.1	0.2	0.1	0.5	0.0	0.0	0.5	0.1
Bacteria	Hyd24-12	-	0.0	0.0	0.0	0.0	0.0	0.0	0.0	0.0	0.0	0.0	0.0	0.0	0.0	0.0
Bacteria	JL-ETNP-Z39	-	0.0	0.1	0.0	0.0	0.0	0.0	0.0	0.0	0.0	0.0	0.0	0.0	0.0	0.0
Bacteria	LD1-PA38	-	0.0	0.4	0.0	0.0	0.2	0.0	0.5	0.1	0.1	0.2	0.0	0.0	0.0	0.0
Bacteria	Lentisphaerae	B01R017	0.0	0.0	0.0	0.0	0.0	0.0	0.0	0.0	0.0	0.0	0.0	0.0	0.0	0.0
Bacteria	Lentisphaerae	LD1-PB3	0.0	0.0	0.0	0.0	0.0	0.0	0.0	0.0	0.0	0.0	0.0	0.0	0.0	0.0
Bacteria	Lentisphaerae	Lentisphaeria	0.0	0.0	0.0	0.0	0.0	0.0	0.0	0.0	0.0	0.0	0.0	0.0	0.0	0.0
Bacteria	Lentisphaerae	Oligosphaeria	0.0	0.0	0.0	0.0	0.0	0.0	0.0	0.0	0.0	0.0	0.0	0.0	0.0	0.0
Bacteria	Lentisphaerae	R76-B128	0.0	0.0	0.0	0.0	0.0	0.0	0.1	0.0	0.0	0.0	0.0	0.0	0.0	0.0
Bacteria	Lentisphaerae	WCHB1-41	0.0	0.4	0.0	0.0	0.1	0.1	1.0	0.0	0.0	0.1	0.0	0.1	0.0	0.0
Bacteria	Lentisphaerae	c5LKS8	0.0	0.0	0.0	0.0	0.0	0.0	0.0	0.0	0.0	0.0	0.0	0.0	0.0	0.0
Bacteria	Lentisphaerae	-	0.0	0.0	0.0	0.0	0.0	0.0	0.4	0.0	0.0	0.0	0.0	0.0	0.0	0.0
Bacteria	NPL-UPA2	-	0.1	0.0	0.0	0.0	0.1	0.0	0.2	0.2	0.0	0.1	0.0	0.0	0.0	0.0
Bacteria	Nitrospirae	Nitrospira	2.9	0.8	0.0	0.0	0.5	1.2	0.2	3.6	0.8	1.0	0.0	0.0	0.0	0.9
Bacteria	Planctomycetes	BD7-11	0.0	0.0	0.0	0.0	0.0	0.0	0.0	0.0	0.0	0.0	0.0	0.0	0.0	0.0
Bacteria	Planctomycetes	MBMPE71	0.7	0.1	0.0	0.0	0.2	0.0	0.2	0.9	0.1	0.5	0.0	0.0	0.0	0.4
Bacteria	Planctomycetes	MD2896-B258	0.1	0.0	0.0	0.1	0.5	0.0	0.0	0.1	0.0	0.2	0.0	0.2	0.0	0.1
Bacteria	Planctomycetes	OM190	0.0	0.1	0.0	0.0	0.1	0.0	0.4	0.0	0.0	0.0	0.0	0.0	0.0	0.4
Bacteria	Planctomycetes	Phycisphaerae	8.6	7.7	1.7	2.5	7.7	5.1	10.3	13.6	1.9	6.5	0.0	6.8	0.0	5.5
Bacteria	Planctomycetes	Pla3_lineage	0.2	0.2	0.0	0.0	0.0	0.4	0.4	0.9	0.0	0.4	0.0	0.0	0.0	0.3
Bacteria	Planctomycetes	Pla4_lineage	0.2	0.1	0.0	0.0	0.2	0.0	0.7	0.0	0.1	0.3	0.0	0.0	0.0	0.0
Bacteria	Planctomycetes	Planctomycetacia	3.9	4.4	0.0	3.0	14.2	0.2	2.7	3.1	2.5	8.2	0.2	0.4	0.7	0.2
Bacteria	Planctomycetes	SGST604	0.0	0.0	0.0	0.0	0.0	0.0	0.0	0.0	0.0	0.0	0.0	0.0	0.0	0.0
Bacteria	Planctomycetes	-	0.6	0.5	0.0	0.1	0.9	0.0	1.7	0.9	0.1	0.7	0.0	0.0	0.0	1.2
Bacteria	Planctomycetes	vadinHA49	0.2	0.0	0.0	0.0	2.0	0.1	0.2	0.2	0.0	0.3	0.0	0.1	0.1	0.0
Bacteria	Proteobacteria	ARKICE-90	0.0	0.2	0.0	0.0	0.0	0.5	0.2	0.0	0.0	0.4	0.0	0.1	0.4	0.0
Bacteria	Proteobacteria	Alphaproteobacteria	3.3	1.1	0.5	6.4	5.2	5.0	1.7	1.3	1.2	2.7	3.8	1.9	2.0	5.4

Bacteria	Proteobacteria	Betaproteobacteria	0.3	0.1	5.4	3.5	0.4	0.0	0.3	0.2	0.0	0.2	0.7	0.8	0.2	1.7
Bacteria	Proteobacteria	Deltaproteobacteria	5.6	12.8	0.2	8.6	7.8	0.9	15.1	3.8	0.5	2.9	0.4	1.4	0.2	2.1
Bacteria	Proteobacteria	Epsilonproteobacteria	0.0	1.7	0.5	0.1	0.0	27.2	4.1	0.0	0.0	4.8	0.0	0.1	0.0	0.8
Bacteria	Proteobacteria	Gammaproteobacteria	7.2	3.8	71.7	57.1	5.1	38.4	14.3	2.9	19.0	12.1	88.2	47.0	91.0	28.8
Bacteria	Proteobacteria	JTB23	0.0	0.0	0.0	0.0	0.1	0.0	0.0	0.0	0.0	0.0	0.0	0.0	0.0	0.0
Bacteria	Proteobacteria	Milano-WF1B-44	0.0	0.0	0.0	0.0	0.0	0.0	0.0	0.1	0.0	0.0	0.0	0.0	0.0	0.0
Bacteria	Proteobacteria	SPOTSOCT00m83	0.0	0.0	0.0	0.0	0.0	0.0	0.0	0.0	0.0	0.0	0.0	0.0	0.0	0.0
Bacteria	Proteobacteria	TA18	0.0	0.1	0.0	0.0	0.0	0.0	0.0	0.0	0.0	0.0	0.0	0.0	0.0	0.0
Bacteria	Proteobacteria	Zetaproteobacteria	0.0	0.0	0.0	0.0	0.0	0.0	0.2	0.0	0.0	0.0	0.0	0.0	0.0	0.0
Bacteria	Proteobacteria	-	0.0	0.3	0.0	0.0	1.2	0.0	0.6	0.0	0.0	0.3	0.0	0.1	0.0	0.0
Bacteria	SHA-109	-	0.0	0.0	0.0	0.0	0.0	0.0	0.1	0.0	0.0	0.0	0.0	0.0	0.0	0.0
Bacteria	Spirochaetae	Spirochaetes	1.6	0.3	0.0	0.9	0.1	0.1	0.9	0.6	1.2	3.1	0.0	0.1	0.0	0.3
Bacteria	TA06	-	0.0	0.4	0.0	0.0	0.2	0.2	0.5	0.0	6.7	1.5	0.0	0.6	0.0	0.0
Bacteria	TM6	-	3.4	0.9	0.0	0.2	2.2	0.0	0.2	0.7	0.1	4.1	0.0	0.0	0.0	0.0
Bacteria	Tenericutes	Mollicutes	0.1	0.1	0.0	0.0	0.0	0.0	0.0	0.1	0.0	0.6	0.0	0.0	0.0	0.0
Bacteria	Verrucomicrobia	OPB35_soil_group	0.0	0.0	0.0	0.0	0.0	0.0	0.0	0.0	0.0	0.0	0.0	0.0	0.0	0.0
Bacteria	Verrucomicrobia	Opitutae	0.0	0.0	0.0	0.0	0.0	0.0	0.0	0.0	0.0	0.0	0.0	0.0	0.0	0.0
Bacteria	Verrucomicrobia	S-BQ2-57_soil_group	0.0	0.0	0.0	0.0	0.0	0.0	0.0	0.0	0.0	0.0	0.0	0.0	0.0	0.0
Bacteria	Verrucomicrobia	Spartobacteria	0.0	0.0	0.0	0.0	0.0	0.0	0.0	0.0	0.0	0.0	0.0	0.0	0.0	0.0
Bacteria	Verrucomicrobia	Verrucomicrobiae	0.0	0.0	0.0	0.0	0.0	0.0	0.1	0.0	0.0	0.0	0.0	0.0	0.0	0.0
Bacteria	WCHB1-60	-	0.0	0.1	0.0	0.0	0.0	0.0	0.0	0.0	0.0	0.0	0.0	0.0	0.0	0.0
Bacteria	unclassified bacteria	-	5.6	3.0	0.5	0.5	15.1	1.2	5.8	4.6	0.9	4.8	0.1	2.2	0.9	1.4
			**100.0**	**100.0**	**100.0**	**100.0**	**100.0**	**100.0**	**100.0**	**100.0**	**100.0**	**100.0**	**100.0**	**100.0**	**100.0**	**100.0**

*nd: no data

Table S3. Nonparametric correlation between the relative abundance of major microbial populations and deeper depths.

	Bacteria					Archaea			
	Gammaproteobacteria	Phycisphaerae	JS1	OP8	Deltaproteobacteria	Thermoplasmata	MBG-B	MG-I	Group C3
Correlation coefficient	0.39	-0.32	-0.09	-0.12	-0.49	-0.06	0.41	-0.36	-0.06
p-value	0.16	0.26	0.77	0.69	0.08	0.84	0.19	0.25	0.84
N	14	14	14	14	14	12	12	12	12

Table S4. Metal contents and DNAs in Core GC1.

Depth[a] (cmbsf)	Metal content (ppm)		DNA content (ng/μl)[b]		
	Mn	Fe	gDNA	Archaea	Bacteria
28	387.85	31922.77		nd	
29	nd[*]		3.57 ± *0.54*	0.0724 ± *0.0089*	0.3708 ± *0.0328*
46	367.67	29245.21		nd	
61	nd		1.29 ± *0.41*	0.0112 ± *0.0001*	0.0451 ± *0.0010*
64	371.5	30215.63		nd	
79	nd		1.22 ± *0.22*	0.0129 ± *0.0023*	0.0285 ± *0.0001*
82	379.48	29035.08		nd	
105	466.12	32047.92		nd	
115	nd		0.59 ± *0.01*	0.0134 ± *0.0020*	0.0289 ± *0.0005*
125	405.86	30591.7		nd	
140	402.16	27436.91		nd	
155	nd		0.86 ± *0.02*	0.0171 ± *0.0007*	0.0552 ± *0.0033*
160	443.84	32009.42		nd	
190	472.68	30524.66		nd	
205	nd		0.39 ± *0.13*	0.0208 ± *0.0057*	0.0280 ± *0.0047*
215	535.52	31575.86		nd	
245	490.47	28842.18		nd	
255	nd		1.28 ± *0.09*	0.0177 ± *0.0016*	0.0428 ± *0.0003*
280	494.91	31496.32		nd	
295	nd		0.81 ± *0.24*	0.0226 ± *0.0006*	0.0222 ± *0.0007*
315	nd		0.93 ± *0.02*	0.0150 ± *0.0001*	0.0210 ± *0.0004*
320	526.58	30308.89		nd	
345	580.23	28009.99		nd	
355	nd		0.59 ± *0.23*	0.0149 ± *0.0006*	0.0102 ± *0.0013*
390	749.56	29134.21		nd	
400	nd		0.41 ± *0.14*	0.0060 ± *0.0001*	0.0657 ± *0.0011*
410	1366.23	30009.57		nd	
445	2657.57	28084.14		nd	
455	nd		0.73 ± *0.46*	0.0076 ± *0.0021*	0.0038 ± *0.0003*
470	2495.86	33827.57		nd	
490	2146.81	34869.84		nd	
505	nd		0.81 ± *0.00*	0.0086 ± *0.0004*	0.0037 ± *0.0003*
520	371.57	26439.14		nd	
540	nd		0.48 ± *0.11*	0.0015 ± *0.0001*	0.0033 ± *0.0004*

[a]1 cm interval sampling in each depth

[b]Values are the average ± standard deviation (italic)

[*]nd: no data

Table S5. Grain texture, water content and accumulation rate of crenarchaeol in the GC1.

Depth (cmbsf)	Grain texture (%)			Water (%)	AC_crenarchaeol (g/cm^2ky)
	Sand	Silt	Clay		
0	0.0	20.9	79.1	44.3	nd*
5				37.5	11.2
10	0.0	21.1	78.9	36.7	9.8
15				37.3	10.0
20	0.0	22.4	77.6	35.3	12.8
25				37.7	12.3
30	0.0	21.5	78.5	37.8	12.5
35				40.6	13.5
40	0.0	17.3	82.7	37.7	12.1
45				41.2	8.7
50	0.0	14.4	85.6	37.8	nd
55				40.8	4.7
60	0.0	20.5	79.5	36.7	nd
65				35.7	nd
70	0.0	21.7	78.4	38.4	10.2
75				35.2	12.8
80	0.0	22.1	77.9	43.1	12.0
85				33.0	11.4
90	0.0	19.9	80.1	37.4	10.6
95				37.3	10.5
100	0.0	21.0	79.1	37.5	nd
105				41.3	11.6
110	0.0	20.0	80.0	39.9	nd
115				41.7	nd
120	0.0	20.8	79.2	41.6	22.4
125				41.9	23.1
130	0.0	20.2	79.9	41.5	19.2
135				42.6	19.1
140	0.0	21.0	79.0	42.4	20.8
145				39.2	22.4
150	0.0	19.8	80.2	41.3	23.5
155				38.9	20.9
160	0.0	20.9	79.1	41.8	17.8
165				41.4	18.4
170	0.0	22.0	78.0	41.4	nd
175				41.1	17.3

180	0.0	19.1	80.9	42.8	24.1
185				42.5	26.0
190	0.0	20.8	79.2	40.3	24.4
195				41.0	27.7
200	0.0	20.3	79.7	40.6	26.5
205				37.9	25.1
210	0.0	16.1	83.9	35.7	25.6
215				40.8	27.5
220	0.0	18.6	81.5	41.8	30.2
225				39.7	25.3
230	0.0	18.9	81.2	38.6	31.6
235				38.5	30.5
240	0.0	20.1	79.9	38.4	30.9
245				38.7	48.7
250	0.0	18.4	81.6	39.2	46.7
255				41.5	nd
260	0.0	20.0	80.0	41.5	45.5
265				41.2	37.8
270	0.0	18.6	81.4	40.2	nd
275				39.6	35.3
280	0.0	18.4	81.6	39.6	41.3
285				35.7	39.0
290	0.0	19.6	80.4	35.6	41.9
295				34.3	41.2
300	0.0	19.4	80.6	36.0	42.8
305				34.2	37.1
310	0.0	17.7	82.3	32.6	40.1
315				31.9	41.2
320	0.0	15.2	84.8	37.4	39.9
325				38.7	42.7
330	0.0	18.1	81.9	34.9	41.7
335				39.3	42.7
340	0.0	21.0	79.0	38.8	43.3
345				39.5	43.0
350	0.0	18.8	81.2	38.0	26.4
355				37.6	27.4
360	0.0	15.5	84.5	37.4	25.4
365				38.7	28.1
370	0.0	19.6	80.4	37.2	29.3
375				38.1	29.3
380	0.0	17.2	82.8	38.0	26.8

385				37.5	29.3
390	0.0	20.6	79.5	36.8	29.3
395				33.6	31.1
400	0.0	13.7	86.3	31.2	31.9
405				33.7	30.0
410	0.0	16.8	83.2	35.5	24.5
415				35.4	24.8
420	0.0	12.1	87.9	35.8	28.4
425				37.5	27.8
430	0.0	13.9	86.1	34.0	27.0
435				36.7	28.3
440	0.0	13.4	86.6	36.2	27.8
445				35.7	27.0
450	0.0	13.9	86.1	34.9	26.9
455				37.4	26.6
460	0.0	13.5	86.6	36.2	18.0
465				36.4	20.9
470	0.0	12.7	87.3	36.6	21.2
475				34.8	21.4
480	1.2	12.5	86.2	36.1	20.1
485				34.7	21.5
490	0.0	13.9	86.1	33.7	21.1
495				33.6	22.2
500	0.0	13.6	86.4	27.3	22.7
505				31.1	24.2
510	0.0	12.2	87.8	29.3	24.2
515				31.7	24.9
520	0.0	13.0	87.1	29.4	24.5
525				27.5	26.4
530	0.0	20.0	80.0	26.0	30.4
535				25.1	30.1
540	0.0	19.6	80.4	27.3	29.7
545				27.2	nd

*nd: no data

Supplementary References

1 Stein, R. *Arctic Ocean Sediments: Processes, Proxies, and Paleoenvironment: Processes, Proxies, and Paleoenvironment*. Vol. 2 (Elsevier, 2008).

2 Kim, J.-H. *et al.* Inferences on gas transport based on molecular and isotopic signatures of gases at acoustic chimneys and background sites in the Ulleung Basin. *Organic geochemistry* **43**, 26-38 (2012).

3 Choi, J. *et al.* Gas origin and migration in the Ulleung Basin, East Sea: Results from the Second Ulleung Basin Gas Hydrate Drilling Expedition (UBGH2). *Marine and Petroleum Geology* **47**, 113-124 (2013).

4 Pimmel, A. & Claypool, G. Introduction to shipboard organic geochemistry on the JOIDES Resolution. (2001).

5 Tamura, K. *et al.* MEGA5: molecular evolutionary genetics analysis using maximum likelihood, evolutionary distance, and maximum parsimony methods. *Molecular biology and evolution* **28**, 2731-2739 (2011).

6 Jakobsson, M. *et al.* The international bathymetric chart of the Arctic Ocean (IBCAO) version 3.0. *Geol. Res. Lett.* **39**, L12609 (2012).
